# Haploinsufficiency networks identify targetable patterns of allelic deficiency in low mutation ovarian cancer

**DOI:** 10.1038/ncomms14423

**Published:** 2017-02-15

**Authors:** Joe Ryan Delaney, Chandni B. Patel, Katelyn McCabe Willis, Mina Haghighiabyaneh, Joshua Axelrod, Isabelle Tancioni, Dan Lu, Jaidev Bapat, Shanique Young, Octavia Cadassou, Alena Bartakova, Parthiv Sheth, Carley Haft, Sandra Hui, Cheryl Saenz, David D. Schlaepfer, Olivier Harismendy, Dwayne G. Stupack

**Affiliations:** 1 Division of Gynecologic Oncology, Department of Reproductive Medicine, UCSD School of Medicine and UCSD Moores Cancer Center, 3855 Health Sciences Drive, La Jolla, California 39216, USA; 2 Centre de recherche en Cancérologie, INSERM 1052, CNRS 5286, Centre Léon Bérard, Université de Lyon, Lyon, France; 3 Division of Biomedical Informatics, Department of Medicine, UCSD School of Medicine and UCSD Moores Cancer Center, 3855 Health Sciences Drive, La Jolla, California 92093, USA

## Abstract

Identification of specific oncogenic gene changes has enabled the modern generation of targeted cancer therapeutics. In high-grade serous ovarian cancer (OV), the bulk of genetic changes is not somatic point mutations, but rather somatic copy-number alterations (SCNAs). The impact of SCNAs on tumour biology remains poorly understood. Here we build haploinsufficiency network analyses to identify which SCNA patterns are most disruptive in OV. Of all KEGG pathways (*N*=187), autophagy is the most significantly disrupted by coincident gene deletions. Compared with 20 other cancer types, OV is most severely disrupted in autophagy and in compensatory proteostasis pathways. Network analysis prioritizes *MAP1LC3B* (*LC3*) and *BECN1* as most impactful. Knockdown of *LC3* and *BECN1* expression confers sensitivity to cells undergoing autophagic stress independent of platinum resistance status. The results support the use of pathway network tools to evaluate how the copy-number landscape of a tumour may guide therapy.

## Abstract (web summary)

Cancers accumulate multiple single copy number alterations, but their impact is unclear. Here, the authors computationally demonstrate a disruption of genes associated with autophagy in ovarian cancer, show impact on autophagic flux, and note the efficacy of autophagy drugs in preclinical models.

Characterization of specific cancer mutations has yielded a map of which oncogenes and tumour suppressors that may be chemically or biologically targetable^1^,^2^ and guided immunotherapy^3^. However, single-nucleotide variants and short insertion–deletion mutations (here referred to simply as ‘mutations') are not the sole drivers of oncogenesis. High-grade serous ovarian cancer (OV) is uniquely low in mutation and high in somatic copy-number alterations (SCNAs). SCNAs drive cancer through losses of tumour suppressors or amplifications of oncogenes, often by large SCNAs encompassing hundreds of genes^4^.

Homozygous deletion occurs rarely (1–2% of SCNAs) due co-deletion of essential genes. On a gene-to-gene basis, SCNAs are more common than mutations even in highly mutated cancer types and ∼95% of SCNAs observed in tumours are monoallelic changes. However, with ∼16,000 genes with SCNAs in the average OV tumour (Fig. 1d), statistical modelling of driver SCNAs is complicated by pervasive ‘background' SCNAs, which may not drive tumour progression. Previous analyses of SCNAs via chromosome arm alterations identified correlated pairs^5^,^6^, but lack a consideration of collaborative monoallelic SCNAs altering entire molecular pathways. Pathway analysis can improve an understanding of which molecular processes are altered when multiple genes contribute to cellular function, since different gene deletion combinations can yield identical phenotypes.

We developed a new tool to analyse highly variable SCNA tumours to determine significantly altered pathways and the gene-level SCNAs, which most likely contribute to pathway disruption. The tool is designed to incorporate known pathway concepts of genetic bottlenecking^7^, and is found to correctly prioritize known tumour suppressors and oncogenes as impactful genes in OV. By this analysis, the most suppressed pathway in OV is autophagy. Many other proteostasis pathways, such as the proteasome, endoplasmic reticulum (ER) stress and the lysosome are suppressed in OV. In validation of these computational findings, treatment of multiple OV *in vivo* models by autophagy- and proteostasis-disrupting drugs abolishes tumour growth. Knockdown of *BECN1* and *LC3B* sensitizes OV to the autophagy halting drug chloroquine. These results implicate autophagy as a major disrupted pathway in OV, which is also amenable to therapy.

## Results

### Half of ovarian tumours lack clear driver mutations

**Figure 1:**
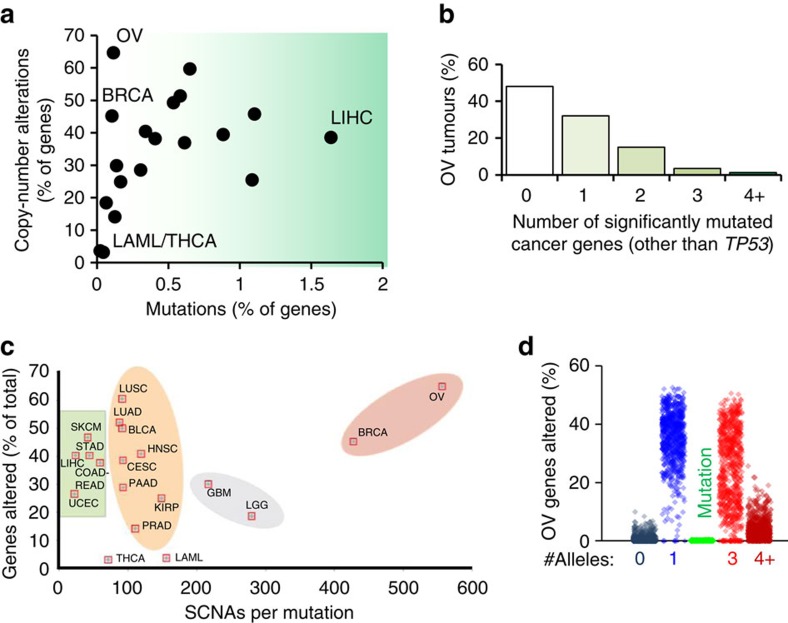
Prevalence of gene-level alterations in cancer. (**a**) The average percentage of genes with either somatic copy-number alterations (SCNAs) or somatic point and small indel mutations for TCGA studied cancers (*N*=9,740 tumors). (**b**) The number of significantly mutated cancer genes^8^ other than TP53 that are mutant in OV is plotted as a percentage of primary tumours from TCGA studied patients. Nearly half have no oncogenic mutation other than TP53. (**c**) Ratio of SCNAs to mutations relative to total percentage of genes changed across cancer types. (**d**) The percent of genes altered by either SCNA (allele numbers 0, 1, 3 or 4+) or by mutation is plotted for each TCGA OV tumour (*N*=579 for SCNAs, *N*=316 for mutations).

OV tumours have been characterized^8^ as being uniquely low in mutations and high in SCNAs (Fig. 1a). However, it is possible that despite relatively low mutation rates, each OV tumour nonetheless contains multiple tumour suppressor or oncogene mutations that drive cancer formation. To investigate this possibility, we analysed The Cancer Genome Atlas (TCGA) OV data for mutations in well-known tumour-driver genes^8^. Interestingly, 48% of studied tumours have no mutations in these oncogenes or tumour suppressors, other than *TP53* (Fig. 1b). Since mutant p53 alone is insufficient for tumour formation^9^,^10^, these tumours likely contain SCNA drivers^5^ which aid in tumorigenesis. Given the high ratio of SCNAs to mutations in OV (Fig. 1c,d), we sought a new method to better understand potential SCNA drivers.

### Design of the HAPTRIG SCNA analysis tool

**Figure 2:**
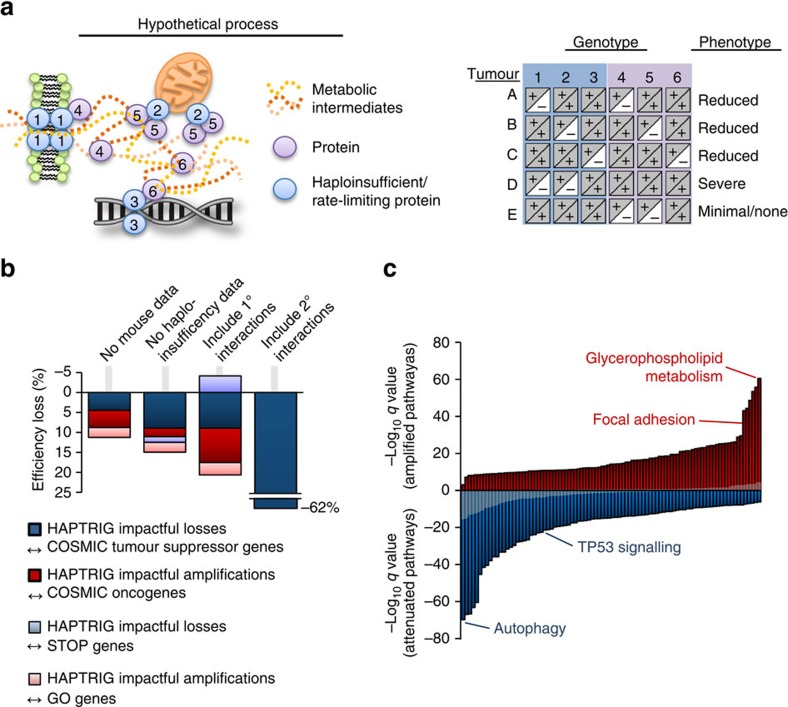
Design of HAPTRIG and OV pan-pathway analysis. (**a**) Schematic of the rationale behind designing HAPTRIG network analyses. Genotypes with similar phenotypes can be spread across many genes and each tumour may alter the phenotype using different genes. Haploinsufficient genes are more likely to drive phenotype changes, as are highly interactive genes. (**b**) Different versions of HAPTRIG were coded and executed to test which inputs prioritized genes with known tumour suppressor or oncogenic function, as annotated in COSMIC, and for ability to prioritize ‘STOP' and ‘GO' genes as expected. HAPTRIG was most effective across all KEGG pathways when considering protein–protein interactions within pathway genes only and when mouse and/or yeast orthologue haploinsufficiency data were included. Including genes that interacted with pathway genes (1° interactors) reduced efficiency as did including genes with an additional interaction distance from pathway genes (2° interactors). (**c**) HAPTRIG network analyses were created for all distinct, human KEGG pathways (*N*=187 pathways) and significantly disrupted pathways are plotted by significance compared with a minimally altered SCNA cancer type, thyroid cancer (THCA; in grey overlay). The top-disrupted pathways are noted in comparison with known canonical OV-disrupted pathways, focal adhesion and p53 signalling. Detailed information on these pathways is in Supplementary Data 1, and secondary OV data sets can be found in Supplementary Data 2 and 3.

We developed a computational tool to identify pathways significantly disrupted by SCNAs in the highly noisy genetic background of OV tumours. The program was designed to analyse diverse genetic backgrounds which all yield at least one similar phenotype (Fig. 2a). Many biological pathways have multiple bottleneck^7^ or regulatory points^11^, any of which can equivalently affect pathway phenotype^12^. While Gene Set Enrichment Analysis (GSEA) also looks at multiple genes within a pathway to determine statistical significance at the cohort level^13^, we designed our tool to incorporate two additional pieces of information to better characterize genetic disturbance of pathway biology: protein–protein interactions (to prioritize genes that modulate other genes within the same pathway) and haploinsufficiency data (to prioritize genes that are known to affect biology when only a single gene copy is altered).

This Haploinsufficient/Triplosensitive Gene (HAPTRIG) tool generates network scores by (1) building protein–protein interaction networks of pathway proteins from BioGRID^14^, (2) prioritizing interactions that contain a haploinsufficient or triplosensitive gene, (3) negatively scoring interactions containing gene deletion SCNAs and positively scoring interactions containing gene amplification SCNAs, and (4) summing all interaction scores within a molecular pathway. For statistical significance, pathway scores from observed tumours were compared with control data of 1000 tumour-paired randomly permutated SCNAs to derive a *P* value of observed tumour pathway changes compared with what would be expected by chance (for a schematic, see Supplementary Fig. 1). This design enables statistically significant pathway changes in a cohort of tumours to be detected in a high noise background. In addition, the HAPTRIG pipeline scores the contribution of each gene within a pathway to allow for ranking the biological importance of each gene within a pathway. For example, since *TP53* is highly interactive and often deleted, it is ranked by the HAPTRIG tool as the most impactful deletion within the p53 pathway for most OV tumours.

To test the robustness of the HAPTRIG approach, we queried HAPTRIG for its ability to prioritize known tumour suppressor genes and oncogenes^15^, as most affecting deleted or amplified gene sets, respectively, and similarly tested for ‘STOP' and ‘GO' gene^4^ prioritization. Using the full HAPTRIG approach as a reference, we measured how its sensitivity is affected by the following parameters: (1) removal of haploinsufficient orthologue data from mice and yeast, (2) inclusion of only intrinsic (within gene set only) interactions or primary/secondary interactions as well, and (3) when gene ontology (GO) pathways were used in place of comparable Kyoto Encyclopedia of Genes and Genomes (KEGG) pathways (Fig. 2b, Supplementary Fig. 2A). All components altered HAPTRIG efficiency in the range of 10–60%. While we predict many GO pathways to be useful in HAPTRIG analysis, GO pathways are typically much larger and contain many genes with tangential relation to core pathway function. The most accurate view of SCNA-altered pathways within OV was thus found by using all distinct, human KEGG pathways (*N*=187 pathways) scored for intrinsic and haploinsufficient interactions.

### HAPTRIG pathway analysis of OV identifies autophagy loss

In TCGA OV cohort, we observed the most statistically unlikely disrupted deletion-enriched pathway to be autophagy (followed by FoxO signalling, adipocytokine signalling, arginine/proline metabolism and NOTCH signalling) and the most statistically unlikely disrupted amplification-enriched pathway to be glycerophospholipid metabolism (Fig. 2c, all disrupted pathway data in Supplementary Data 1). Known altered pathways such as p53 and focal adhesion were also significantly altered, albeit at lower significance. This pattern persisted in an independent OV cohort^16^ but did not reach statistical significance in an endometrioid OV cohort, perhaps due to small sample size (Supplementary Fig. 2; Supplementary Data 2 and 3). While we focus on KEGG pathways here, HAPTRIG functions on any pathway set (Hallmark pathway set results shown in Supplementary Data 4). HAPTRIG improves on GSEA to identify these significantly disrupted pathways: only two KEGG pathways reached statistical significance using GSEA (Supplementary Table 1; Supplementary Fig. 3). We release the code for HAPTRIG as Supplementary Software 1, and provide example input data sets as Supplementary Data 5.

**Figure 3:**
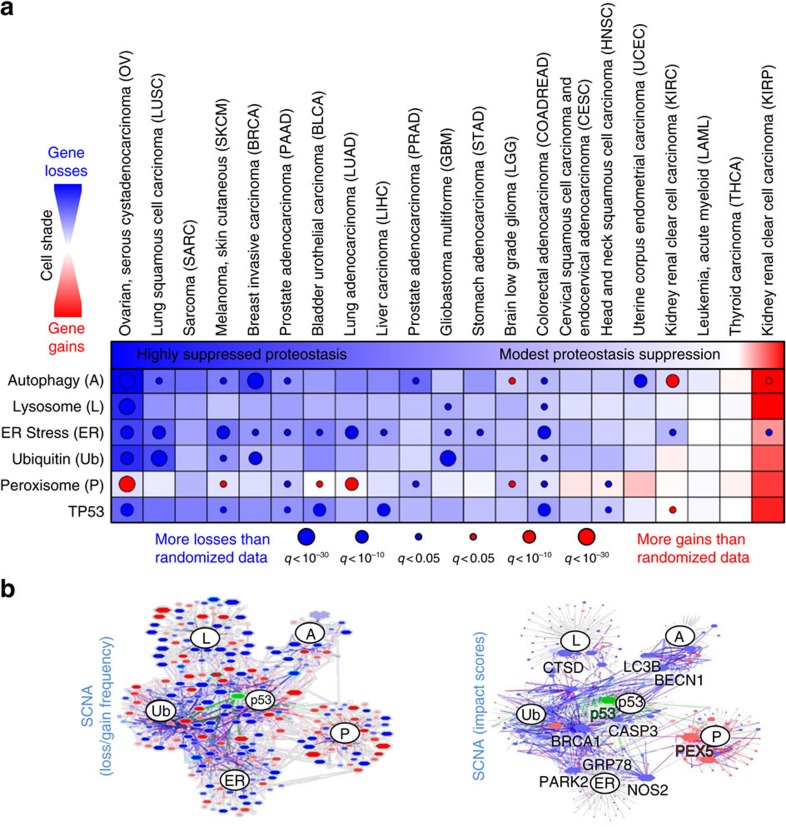
Summary of HAPTRIG proteostasis network scores across 21 cancer types. (**a**) HAPTRIG analyses were performed for proteostasis pathways and the p53 pathway. Since these pathways are functionally interdependent, HAPTRIG scored both intrinsic and primary interactions from within these different pathways. The chart displays pathway network scores as blue fill if deletion-enriched, red fill if gain-enriched, and white fill for neither. Significance is represented as overlaid circles of size proportional to the log_10_ *q* value. (**b**) OV HAPTRIG networks were visually graphed by Cytoscape, with gene node and edge protein–protein interaction size proportional to the penetrance of the gene changes within the cancer type (left panel) or by HAPTRIG predicted gene-impact scores (right panel). A red fill is assigned if the majority of copy-number changes are positive, and blue if they are negative. Node outlines are highlighted in cyan if haploinsufficiency annotations are associated with that gene. Green fill and edges indicate genes mutated in >10% of the tumour cohort. Expanded HAPTRIG OV networks, with gene labels, are available in Supplementary Fig. 3.

Autophagy has long been implicated in tumour development and may have dual roles: loss of autophagy genes including *BECN1* leads to early oncogenesis in mouse models^17^,^18^; however, *KRAS* mutant cancers are addicted to elevated autophagy^19^. Interestingly, most proteostasis pathways in our pan-pathway analysis were enriched for deletions, including ER stress, ubiquitin-mediated proteolysis and the lysosome, although the peroxisome pathway was enriched for amplifications. Haploinsufficiency in model organism screens is associated with an inability to form adequately proportioned protein–quality control complexes^20^, suggesting single allele SCNAs disrupt these pathways. To determine whether proteostasis disruption was specific to OV, we ran HAPTRIG analyses across 20 other cancer types studied by TCGA. Alterations ranged from minimal among acute myeloid leukaemia and thyroid cancers, a strongly suppressed network of proteostasis genes in invasive breast (BRCA) and serous ovarian (OV) cancers, to a uniquely amplified autophagy network in renal papillary cell carcinoma (KIRC; Fig. 3a). Many genes were frequently altered in OV, and HAPTRIG ranked known biologically impactful genes (for example, *BRCA1*, *TP53*, *BECN1* and *CASP3*) as most altered for OV (Fig. 3b, full OV networks in Supplementary Fig. 4), as well as some genes uncommonly associated with cancer (for example, *CTSD* for lysosomal function and *PEX5* for peroxisomal function, full summary in Supplementary Table 2). OV was clearly the most disrupted for proteostasis amongst these 21 tumour types. We next evaluated whether these SCNA network alterations contribute to cancer phenotypes as mutations do, and whether they might be predictably targeted.

### Targeting autophagy and proteostasis *in vivo* halts OV growth

Well-controlled single-allele losses reduce messenger RNA (mRNA) expression up to 90% of the time, even in a single unstressed experimental condition^21^. In OV, protein expression correlated with mRNA expression for 80–90% of genes^22^. Autophagy depends on mRNA induction for full function^23^. TCGA OV tumours exhibit decreased mRNA expression of core autophagy genes upon heterozygous loss and often contain several core autophagy gene deletions (Supplementary Fig. 5). Such pervasive deletions in protein and organelle quality control genes may sensitize OV to proteotoxic, autophagy-stressing drugs^24^; redundant losses may underlie the severely compromised capacity of these tumours to compensate for proteotoxic treatment combinations (Supplementary Fig. 6). To investigate this possibility, we treated OVCAR3 cells with chloroquine, to prevent autophagy resolution^25^, and nelfinavir, to promote ER stress^26^. Protein aggregates increased by 3–6-fold (Supplementary Fig. 7), concurrent with the accumulation of autophagolysosomes (Supplementary Fig. 8). The phenotype was further amplified when chloroquine/nelfinavir was combined with rapamycin and/or dasatinib^24^, which we term Combination Of Autophagy Selective Therapeutics (COAST; Supplementary Fig. 8). Proteasomal inhibitors also stress autophagy, and bortezomib exhibited cytotoxicity in the low nanomolar range. However, bortezomib was not OV selective and risks high clinical toxicity (Supplementary Fig. 9). Cytotoxic concentrations required for the OV tumour cells were low for other proteostasis-targeting agents (Supplementary Figs 10 and 11). Chloroquine and nelfinavir within the concentration range found in patients' blood^24^ was sufficient to prevent single-cell colony formation, cell growth in suspension, and to promote cytotoxicity (Supplementary Fig. 12) in OV cells. Higher-order combinations (COAST) were selective across six different OV tumour cell lines (Supplementary Fig. 11) with autophagy gene deletions (Supplementary Table 3), and no drug or combination reduced the effects of any other drug.

**Figure 4:**
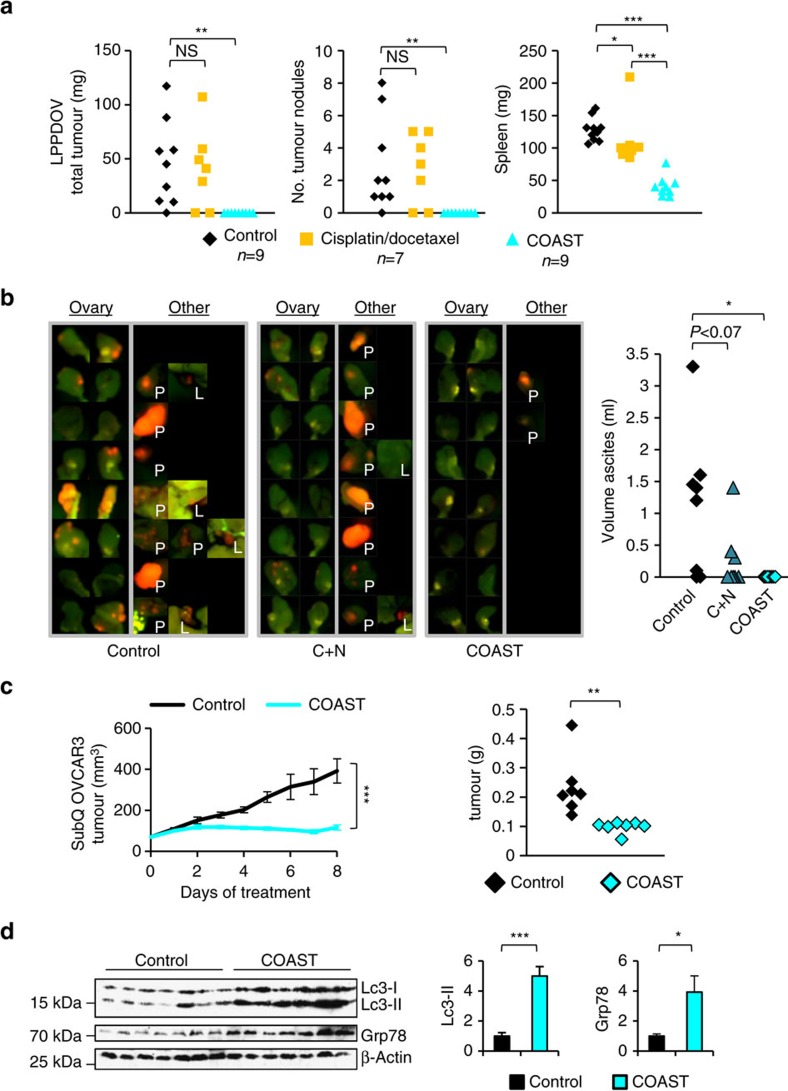
OV tumours are sensitive to disruption of proteostasis. (**a**) Low passage patient-derived OV (LPPDOV) ascites cells from a patient who failed cisplatin–docetaxel chemotherapy were injected i.p. into Nu/nu mice, allowed to disseminate and grow for 10 days, and then treated with control 50% PEG400 or with COAST (Combination of Autophagy Selective Therapeutics: chloroquine 30 mg kg^−1^, nelfinavir 250 mg kg^−1^, rapamycin 2.24 mg kg^−1^, dasatinib 4 mg kg^−1^ and metformin 150 mg kg^−1^ in 50% PEG400) daily for 15 days. An additional control group was treated with cisplatin/docetaxel chemotherapy (injected i.p. with 1 mg kg^−1^ cisplatin and 2.5 mg kg^−1^ docetaxel once per week starting at the first control treatment day for 2 weeks). Upon harvest, all visible and palpable tumours in the peritoneum space were dissected, counted and weighed, as were mouse spleens. (**b**) C57BL/6 immunocompetent mice were injected i.p. with ID8-IP-mCherry cells (*N*=8 per group). After 2 weeks to permit tumour establishment, mice were orally gavaged daily with control 50% PEG400, with COAST, or chloroquine and nelfinavir alone. At 14 days, control mice developed ascites. All groups were killed, ascites were measured and tumour burden assayed by native mCherry fluorescence. Ovaries are displayed for all mice, and any additional tumor fluorescence observed is displayed on the right panel with labels ‘P' for peritoneal wall growth and ‘L' for liver. (**c**) Nu/nu mice with 100 mm^3^ subcutaneous OVCAR3 tumours were gavaged with COAST or control and tumour growth monitored by digital calipers for 7 days. Tumours were then dissected, weighed and (**d**) subjected to immunoblotting for autophagosomal Lc3-II and the ER stress marker Grp78 (mean±s.e.m. *N*=7 mice per group). *P<0.05, **P<0.01, ***P<0.001 by Wilcoxon rank-sum test.

We next evaluated whether this HAPTRIG-informed choice of drugs would ameliorate disease in preclinical models of OV. Cisplatin and docetaxel did not alter the growth of a patient-derived xenograft model derived from a recurrent chemotherapy-resistant patient (Fig. 4a), while the proteostasis-targeted cocktail resulted in a striking complete ablation of tumour growth. Given the lack of any macroscopic disease, we next used an ID8-IP-mCherry labelled tumour model^27^ to allow detection of persistent microscopic disease. Again, mice treated with COAST showed eradication of tumours, although microscopic nests of cells were still detected in 2/8 mice. Interestingly, chloroquine and nelfinavir alone did not result in statistically significant inhibition (Fig. 4b), despite having the best efficacy of two drugs *in vitro* (Supplementary Fig. 10), possibly reflecting the complexity of the tumour microenvironment and other forms of heterogeneity in syngeneic models. This five drug cocktail was remarkably well tolerated in mice^24^, in which we tested up to 8 weeks of COAST therapy, long after all control mice perished (Supplementary Fig. 13). COAST also arrested tumour growth in a subcutaneous OVCAR3 model (Fig. 4c), with residual tumour showing accumulation of autophagosomal Lc3-II and the ER stress marker Grp78 (Fig. 4d).

### Impactful HAPTRIG genes influence OV drug targeting

**Figure 5:**
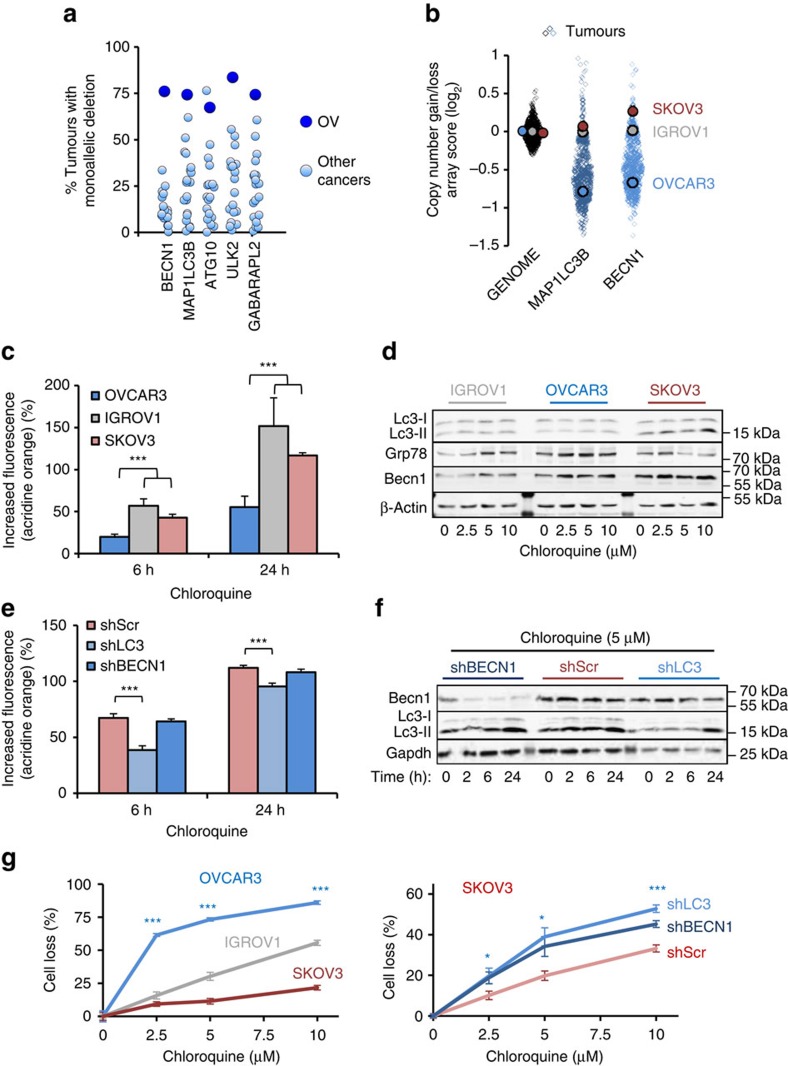
Suppression of *LC3* and *BECN1* lowers cellular capacity to overcome proteotoxicity. (**a**) The five genes most lost in the autophagy KEGG pathway in OV compared with 20 other cancers in tumour gene loss prevalence. (**b**) Log_2_ SNP6 array scores for each tumour in OV compared with three OV cell lines. ‘Genome' corresponds to the average gene score for an individual tumour. OVCAR3 is the most established OV cell line with high-grade serous genetics^30^, whereas IGROV1 and SKOV3 are ovarian cancer cell lines without serous OV genetics. (**c**) OVCAR3 has delayed accumulation of acidic vacuoles including autophagosomes and lysosomes, as measured by acridine orange flow cytometry, when treated with the autophagy/lysosome inhibitor chloroquine (10 μM). Data represent the mean±s.e.m. from four independent experiments. Note that additional cell lines are tested in Supplementary Fig. 15. (**d**) Western blots of autophagosomal Lc3-II indicate reduced accumulation of autophagosomes in OVCAR3 and increased levels of ER stress marker Grp78 when treated with chloroquine. Lysates from three independent experiments were analysed and a representative blot is shown. (**e**) SKOV3 cells knocked down by *BECN1* and *LC3* shRNA were treated with 10 μM chloroquine for the indicated times. Only *shLC3* showed reduced accumulation of autophagosomes by flow cytometric reading of acridine orange stain. Data represent the mean and s.e.m. from four independent experiments. (**f**) Western blots of cells treated as in e, showing reduced Lc3-II accumulation only in *shLC3* cells. Lysates from three independent experiments were analysed and a representative blot is shown. (**g**) OV cells were treated with chloroquine for 48 h at the doses indicated and stained for cell loss by crystal violet. Data represent the mean±s.e.m. from eight independent experiments. *P<0.05, ***P<0.001 by two-tailed Student's *t*-test.

Since genetic targeting is an important consideration of new therapies, we next utilized HAPTRIG network information to determine gene SCNAs most likely to have an impact on autophagy in OV. These most ‘impactful' genes were identified by summing the score contribution of each gene within HAPTRIG networks across all tumours. We ranked impactful suppressive and oncogenic genes for all pathways in OV (Supplementary Table 2). For autophagy, the two highest impact genes were *MAP1LC3B* (*LC3*) and *BECN1*. These two genes were also commonly lost in OV, along with *ATG10*, *ULK2* and *GAPARAPL2* (Fig. 5a). *LC3* and/or *BECN1* are monoallelically deleted in 94% of OV (Supplementary Fig. 5C). Mechanistically, this may explain the sensitivity of OV tumours to drugs pressuring the autophagy network, since orthologues of each gene confer haploinsufficiency in yeast^20^ or mice^18^. These losses occur early in the evolution of OV^28^ and have an associated defect in expression when monoallelically lost (Supplementary Fig. 5), consistent with previous reports^29^. OV cell lines that differ in *LC3* and *BECN1* gene dose (Fig. 5b) were next tested for differences in autophagy.

OVCAR3 is a cisplatin-resistant tumour cell genetically similar to TCGA assayed OV^30^, exhibiting monoallelic deletions of *LC3* and *BECN1*, and forming appropriate high-grade histology in mice^31^. In contrast, IGROV1 and SKOV3 are characterized as ovarian, but not serous (nor high SCNA) ovarian, cancer^30^ cell lines that have lost neither allele (Fig. 5b). Flux through autophagy showed a delayed response in OVCAR3 relative to IGROV1 and SKOV3 following treatment with chloroquine, as measured using complementary assays (Fig. 5c,d)^25^,^32^. Similar results were found when autophagy was perturbed with rapamycin, nelfinavir or combination (COAST) treatments (Supplementary Fig. 14). While few OV cell lines are currently well established and also contain common OV genetics^31^,^33^,^34^, we additionally studied OVCAR5, OVCAR8, the patient-derived xenograft model cells LPPDOV and A2780 for autophagic response to chloroquine and again found cell lines with low HAPTRIG scores to poorly induce autophagy upon chloroquine stress (Supplementary Fig. 15), which correlated with increased cell death. Taken together, although OV cells are not completely lacking autophagy, a maximized response to stress is compromised among cells with losses in autophagy genes such as *LC3* and *BECN1*.

To test directly whether suppression of *LC3*/*BECN1* was sufficient to confer a proteostasis bottleneck, we next evaluated IGROV1 or SKOV3 cells stably expressing lentiviral shRNA selected for modest suppression (∼35–70%) of *LC3* or Beclin. Slowed autophagosome accumulation was clearly observed with *shLC3*, although not significantly with *shBECN1* (Fig. 5e,f). Cells with reduced *LC3* or *BECN1* showed compromised survival following treatment with chloroquine, which prevents clearance of autophagosomes^35^ (Fig. 5g). This survival defect was observed with multiple cell types, including IGROV1 and a glioblastoma (U373) resistant to autophagy drugs (Supplementary Figs 10 and 16). Resistance to cisplatin, a standard of care agent used to treat OV, was not indicative of response to COAST drugs including chloroquine (Fig. 5g; Supplementary Figs 10 and 15). Rather, autophagy-stressing drugs compromised cell survival selectively among lines with autophagy gene losses, regardless of single or combined drug treatment (Supplementary Figs 10, 11 and 15). The results support a model implicating haploinsufficiency, at a minimum for *LC3* and *BECN1*, in the sensitivity of OV to agents targeting autophagy.

## Discussion

The HAPTRIG tool represents an initial haploinsufficiency network-based analysis program that can be applied genome wide for any cancer. Sequencing of mutations has identified potentially targetable genes in minorities of OV patients^34^,^36^. However, given the excessive (two-third of the genome) SCNAs present in OV (Fig. 1a–d), we undertook a strategy to identify pathways that are uniquely and perhaps unexpectedly disrupted by SCNAs. Our permutation strategy enabled identification of significant pathways despite a potentially passenger-filled SCNA landscape. Critically, aside from merely identifying known altered genetics such as suppression of the p53 pathway, enhancement of the focal adhesion pathway and disruption of homologous recombination repair pathways^37^, our top hits are not currently considered to be canonical OV driver pathways. Yet, using *in vivo* and *in vitro* models, we validated that autophagy was suppressed in OV and moreover that by targeting this suppression by drugs that disrupt proteostasis we achieved remarkable tumour remission independent of platinum resistance.

Given the strong autophagy phenotypes we found in OV, it is curious why the autophagy pathway has not been emphasized in prior integrative analysis publications. Previous publications have supported the finding that OV is deficient in DNA repair pathways, dysregulated in cell cycle control and often overexpress MYC and ERBB2 (Supplementary Table 4). HAPTRIG confirms these disruptions in KEGG pathways and in MSigDB (Molecular Signature Database) Hallmark pathways. Interestingly, GSEA^13^ of copy-number data also highly ranks these pathways and autophagy, albeit at a lower rank than HAPTRIG. This is likely because GSEA does not incorporate interaction or haploinsufficiency data, resulting in an altered spectrum of prioritized genes relative to HAPTRIG. A second significant reason that autophagy has not received further exposure in the context of OV is that very few pathway sets include autophagy. In the many thousands of pathways annotated in MSigDB^38^, autophagy is only included in KEGG and GO pathways, as assayed here. Many genes remain to be annotated within pathways^39^, and improved pathway curation will certainly advance pathway analysis tools such as HAPTRIG.

Although loss-of-heterozygosity accompanied by mutations is a recognized phenomenon in breast, ovary, and other cancer, 99.8% of gene deletions in OV show no mutation in the opposing allele. For autophagy genes, mutations in the remaining allele for tumours with heterozygous deletion were not observed. Rather, cumulative gene expression changes from SCNAs contribute to biological phenotypes^40^,^41^,^42^. Reduced gene expression is observed much more commonly than no change in controlled heterozygous deletions^21^, and mRNA correlates with protein expression in ∼80–90% of OV mRNAs^22^. Losses of proteostasis genes are likely oncogenic; multiple studies implicate *BECN1* as a haploinsufficient tumour suppressor in mice^17^,^18^, possibly related to roles in chromosomal segregation during cell division^43^,^44^. Chromosome instability in human cancers such as OV and BRCA may be further exacerbated by loss of *BRCA1*, a functionally independent tumour suppressor neighbouring *BECN1* (ref. 45) on cytoband 17q21. Early losses in autophagy genes may contribute to the extreme SCNA heterogeneity of OV, but as we have shown here, also provide opportunity for network-targeted therapy.

The prevalence of such monoallelic changes has been largely unappreciated. In all cancer types, more genes are affected by single gene-dose changes than by biallelic deletions, doubling or more amplifications, and mutations combined. Tumour selection for specific chromosomal arm losses or duplications follow enrichments for tumour suppressors or oncogenes, respectively^4^,^41^. However, methods to interpret effects and implement action on SCNAs have been underdeveloped. Monoallelic SCNAs may sometimes be viewed as a gene-dose equivalent of a passenger mutation, but scoring collaborative and cumulative pathway interactions and alterations and comparing to a permuted control enabled HAPTRIG to sort through this ‘passenger' noise and yield significant results. We developed the HAPTRIG tool to accurately predict targetable individual gene losses for the autophagy pathway in OV, and have further provided quantitative predictions for all disrupted OV pathways (Supplementary Data 1,2,3,4). In addition, we have provided a free web-tool (https://delaney.shinyapps.io/HAPTRIG_Single_Module_Beta/) to allow the community to easily perform a HAPTRIG analysis on 21 cancer types with 187 unique KEGG pathways.

We suggest that a roadmap of targetable genetic changes in tumours need not be limited to mutations, and HAPTRIG may therefore reveal additional targetable pathways across cancer types. COAST therapy should be clinically tested in OV, given its strong effects, minimal toxicity^24^, and genetic rationale.

## Methods

### HAPTRIG analysis construction

HAPTRIG proteostasis networks were built from the KEGG pathways autophagy (hsa04140), Lysosome (hsa04142), endoplasmic reticulum processing (hsa04141), ubiquitin-mediated proteolysis (hsa04120), peroxisome (hsa04146) and the p53 (hsa04115) pathway. The KEGG autophagy pathway was further curated using current knowledge by adding *MAP1LC3B*, encoding the protein most commonly used to define autophagosomes^25^. We used protein–protein interactions (PPIs) from the BioGRID curated database^14^ to connect input pathway genes. For the pan-pathway analysis and in quality control networks, all human KEGG pathways were used. The full list of 187 KEGG pathways tested is included in Supplementary Data 1.

We obtained copy-number data (*N*=579 tumours for OV) from the UCSC cancer genome Browser^46^, using copy-number calls from the GISTIC2.0 algorithm^47^. For the 2009 OV data sets^16^, log_2_ segmented copy-number data were used, since the array used was not a SNP6 array. There were 102 serous tumours and 11 endometrioid tumours.

To incorporate information regarding dose sensitivity of genes into our network scores, orthologous data sets were used. Yeast data were extracted (17 August2015) from YeastMine^48^, with the query ‘Phenotype=Haploinsufficient' or ‘Phenotype=Haploproficient'. Similar annotations for 169 murine genes were extracted (9/17/2015) from the Mouse Genome Informatics database or the MouseMine database^49^. Human homologues for mouse and yeast genes were systematically determined using the ‘Homology' tool of MouseMine and YeastMine. Of the 486 proteostasis genes studied, 284 were annotated as gene-dose-sensitive. All gene annotations can be found in Supplementary Table 3.

Each edge connecting two gene nodes was scored for negative (loss or deletion) or positive (gain or amplification) copy-number change as follows. Given an edge between gene1 (G1) and gene2 (G2), edge scores were calculated as:

For either (G1,G2) GISTIC<0 (at least one gene is deleted):


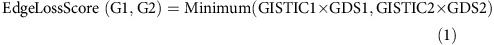


For both (G1,G2) GISTIC≥0 (neither gene is deleted):


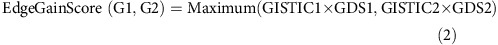


Wherein GISTIC scores represent a range of (−2, −1, 0, 1, 2) from −2 as a double deletion, −1 as a monoallelic deletion, 0 as no somatic change, 1 as a monoallelic gain and 2 as a gain of two or more alleles, and gene dose sensitivity (GDS) indicates the gene-dose sensitivity information (1 for no information, 2 for yeast information and 3 for mouse information).

For Fig. 2, the pan-pathway analysis utilized only gene edges within the given pathway (for example, only genes within the autophagy pathway). For Fig. 3, wherein interactions between proteostasis pathways were important to consider, edges were also utilized in the analysis if one gene in the edge contained a gene in another proteostasis pathway.

For each pathway within a cancer type, we first calculated for each patient the sum of edge scores. We then normalized to the minimum possible haploinsufficient score of that module (a score in which every gene within the module had a monoallelic loss). We further average these normalized scores across all tumours within a TCGA cohort to produce the colourized depiction of average network score suppression (blue) or enhancement (red) in Fig. 3a.

Each cancer type has a unique distribution of chromosome losses and gains. Since a highly copy-number variable cancer may have a higher chance of a random loss or gain of a pathway than a relatively SCNA stable cancer, we compared the distribution of observed HAPTRIG module scores to that of the distribution of HAPTRIG module scores resulting from globally shuffled gene copy-number data from the same cancer cohort (Supplementary Software 1). Edge scores were then recreated using the shuffled gene data. Two distributions for each cancer type were thus created using identical calculations: an observed HAPTRIG module score distribution corresponding to observed tumour data, and a statistical comparison HAPTRIG module score distribution corresponding to randomized data To increase the confidence in the output *P* value, our automated HAPTRIG code creates 1,000 control network scores for each tumour and output *P* values are generated from the average log_10_(*P* value) resulting from these 1,000 control network comparisons. HAPTRIG score distributions were compared by Student's *t*-test and multiple hypothesis testing corrected by the Bonferroni method (for 6 pathways and 21 cancer types=126 comparisons in Fig. 3, 187 comparisons—all KEGG pathways—for Fig. 2) to generate a q value.

Visual networks were drawn using Cytoscape 3.3 (ref. 50). To produce a representative network for the entire OV cohort, the EdgeScores were recomputed at the cohort level using mean GISTIC scores across all tumours. If a node had an SCNA alteration in >33% of patients, an edge was drawn to its PPI partner (blue: loss, red: gain, purple: antagonistic). To accommodate lower numbers of mutations relative to SCNA events, if a gene reached a mutation rate of above 10%, PPI edges were represented as disrupted by mutations (green edge visualization). Node size and colour represent their frequency of SCNAs: blue for more common losses, red if for more common gains, and green if mutated in >10% of patients. Node shade represents the prevalence of the most frequent SCNA event. Node outlines are coloured bright cyan if mouse GDS information was incorporated, and light cyan if yeast GDS information was incorporated. Grey edges depict associations of genes with their respective KEGG molecular pathways.

For gene-impact prioritization, EdgeScores were summed among all tumours within a cohort. Scores were then summed for each gene within the proteostasis network (the gene could be on either end of the edge). The sum of scores was used to rank those genes which had the lowest values (genes of highest network score impact for losses) as well as rank those genes that had the highest values (genes of highest network score impact for gains). A summary of the highest and lowest scoring five genes for each KEGG molecular pathway is provided in Supplementary Data 1.

For quality control, a table of the top 10 ranked genes (as in the gene-impact prioritization) for each of the 187 KEGG pathways was generated and compared with the appropriate COSMIC tumour suppressor/oncogene gene set or STOP/GO gene set. Efficiency was calculated as the per cent of possible hits that were found to be present in the quality control table.

### Code availability

Complete HAPTRIG code is available as Supplementary Software 1. Demo data for input are provided as a convenience as Supplementary Data 5.

### Gene set enrichment analysis

TCGA OV data were used as the expression data set, with tumour copy number compared with normal tissue control copy number. Gene sets were the same as HAPTRIG. Gene set permutations were set at 1,000. To find oncogenic pathways, the comparison was TUMOR_versus_NORMAL, to find tumour suppressor pathways, the comparison was NORMAL_versus_TUMOR. Leading edge analysis was performed and the top 10 genes for each pathway were input as benchmarking genes for quality control analysis, as described above. GSEA version used was 2.2.2.

### Cell culture and reagents

Established cell lines were purchased from the American Type Culture Collection and validated by short tandem repeat profiling (Promega). Routine microscopic morphology tests were performed before each experiment. Cells were verified to be mycoplasma negative by a PCR assay (Agilent Technologies (Stratagene), cat# 302008). Patient consent was obtained for scientific use and publication of the LPPDOV patient-derived OVs, as previously described^24^. All cells were grown in RPMI (Life Technologies) supplemented with 2% glucose, nonessential amino acids (Mediatech #45000-700), sodium pyruvate (Mediatech #45000-710), antibiotics (penicillin, streptomycin and amphotericin, Mediatech #30-004-CI) and 10% fetal bovine serum (Omega Scientific #FB-11). Cells were cultured at 37 °C with 5% CO_2_.

*Antibodies*. All primary antibodies were used at 1:1,000 dilution. LC3B (Novus Biologicals #NB100-2220), p62 (BD Biosciences #610382), β-actin (Sigma-Aldrich #A5441-.2ML), GRP78 (BioLegend #644402), BECN1 (SantaCruz sc-11427), PIK3C3 (Abgent AP1851b), GABARAPL2 (Abgent AP1822d), ATG5 (Cell Signaling 8540P), γ-tubulin (Sigma-Aldrich T6557), GAPDH (GeneTex #239) and DyLight secondary (1:15,000 dilution) antibodies were used: 800 nm for anti-rabbit (VWR #PI35571) and 680 nm for anti-mouse (VWR # PI35518). Secondary horseradish peroxidase antibodies were anti-rabbit (Jackson ImmunoResearch #211-032-171) anti-rat (Life Technologies #619520) or anti- mouse (Jackson ImmunoResearch #115-035-003).

*Drugs*. Docetaxel (Winthrop, US, 20 mg ml^−1^ injection concentrate) and cisplatin (Teva Pharmaceuticals, US, 1 mg ml^−1^ injectable) were obtained by the Moores Cancer Center pharmacy. Metformin (VWR, cat# 89147-892), rapamycin (LC Labs, cat# R-5000), dasatinib (LC Labs, cat# D-3307) and nelfinavir (Creative Dynamics Inc, special order, or for *in vivo* studies Viracept, Agouron Pharmaceuticals) were purchased in powdered form.

*Knockdown shRNAs*. Knockdowns for *MAP1LC3B* and *BECN1* were purchased from ThermoFisher Scientific (#RHS4533-EG8678). At least two shRNAs were always used to generate the presented figures. PEG400 for *in vivo* drug vehicle was from Spectrum Laboratory Products (#TCI-N0443-500G).

### Transmission electron microscopy

Three million cells were seeded onto 10 cm tissue culture (TC) plates, grown for 24 h and then treated with control dimethylsulphoxide/water, nelfinavir (10 μM), chloroquine (10 μM) or COAST (which includes metformin, 10 μM, chloroquine, 10 μM, nelfinavir, 10 μM, rapamycin, 10 nM and dasatinib, 50 nM. Supernatant was removed at 12 h, 10 ml fixative added and incubated at room temperature for 10 min, and then samples were immediately processed by our electron microscopy core. For the analysis, pictures were blinded and then scored using ImageJ to quantify regions of protein aggregates, as measured by high electron density.

### Statistics

In all figures, *P<0.05, **P<0.01, ***P<0.001. *In vivo* tests used Wilcoxon rank-sum with the exception of live subcutaneous tumour measurements, which was tested by analysis of variance two factor with replication (a *t*-test of tumour sizes reaches *P*<0.05 at day 2). All other *P* values were calculated using a two-tailed Student's *t*-test unless otherwise noted. All experiments were performed at least three times with combined data quantified and representative images shown, with the exception of mouse and electron microscopy experiments that were performed once. For HAPTRIG tool statistics, refer to HAPTRIG section above.

### *In vitro* growth inhibition and death assays

Assay data are from at least four independent experiments. If shRNAs were used, with two or more shRNAs per gene were always tested. A total of 2.5–5k cells were seeded onto 96-well TC-treated plates, allowed to adhere for 30 min and then treated with drugs or control vehicle for a total volume of 100 μl. Plates were placed at 37 °C for 48 h unless otherwise indicated. Media was removed and cells were washed once with 125 μl PBS. PBS was then removed and 50 μl crystal violet stain (0.11% crystal violet, 0.17 M NaCl, 22% MeOH, in water) was added. After 30 min room temperature staining, stain was removed and 125 μl PBS was added as a wash. Supernatant was carefully removed to minimize cell disturbance but maximize removal of unspecific crystal violet. Plates were then dried at 37 °C for 1 h without lids and 85 μl MeOH was added to solubilize the crystal violet. Absorbance was read at 600 nm to determine cell density, and background was subtracted. Per cent cell loss was calculated using the formula: 100−(100 × AbsDrug/AbsControl), which incorporates both slowed growth as well as dead cells.

For specificity calculations in Supplementary Fig. 11, the average growth inhibition of U373 and IGROV1 is subtracted from the average growth inhibition of OVCAR3, 5, 8, 10 and LPPDOV to yield the average per cent difference in growth inhibition between groups, which is termed the Specificity % in the graphs. For Supplementary Fig. 11C, the 17 drug combinations including the labelled drug from Supplementary Fig. 11A were used to obtain a ‘Drug Landscape Specificity'. This calculation was: Drug Landscape Specificity=log_2_(Survival(U373)/Survival(CellLineX)), where survival is the average survival of the 17 drug combinations and CellLineX is one of the OV lines.

For soft agar assays, 0.5% agar/RPMI layer was laid by pipetting 50 μl agar into wells of a 96-well plate. The top layer contained 500 cells per 50 μl, in 0.3% agar/RPMI. After agar solidified, drugs were added with another 50 μl of agar-free RPMI. After 7 days of growth, colonies were stained by 0.005% crystal violet, imaged and analysed for size by ImageJ. To determine number of cells per colony, a duplicate plate was stained immediately after seeding to provide images of single cells. Colony sizes were assumed to be spherical to calculate the number of constituent cells.

For suspension assays, cells were seeded to 100k cells per 4 ml RPMI with or without drug and grown in six-well polyHEMA plates. After 3 days of growth, cells were spun down (500 g, 5 min), washed in PBS, trypsinized 5 min, spun down and washed in PBS again, and then stained by trypan blue to obtain viable single-cell counts via a Vi-Cell XR automated cell counter (Beckman Coulter).

### Autophagic flux microscopy

OVCAR3 cells with mCherry-GFP-LC3B virally integrated were seeded on a glass bottom 12-well plate to 5,000 cells per well and treated with COAST drugs (chloroquine (10 μM, C), nelfinavir (10 μM, N), rapamycin (R, 10 nM) and dasatinib (D, 50 nM)). Cells were then imaged live by a Olympus XI-51 spinning disc microscope fitted with an environmental chamber set to standard 5% CO_2_ 37 °C conditions.

### Western blotting

Cells were grown to 50% confluency on 10 cm plates and treated with drugs or control for 24 h at 37 °C. Media was collected, cells washed in PBS and the supernatant was spun 500 g. Iced RIPA buffer (supplemented with a protease inhibitor cocktail (Sigma-Aldrich), 2 mM sodium orthovanadate and 50 mM NaF) was added to solubilize the cells (15 min, room temperature) at which point cells were collected using a cell lifter (Fisher Scientific). Supernatant cells were added to the RIPA buffer and combined with adherent cell fraction. Lysates were spun at 10,000*g* for 10 min at 4 °C, and supernatant was saved and quantified by bicinchoninic acid (BCA) assay (Pierce #23235). A measure of 30 μg of protein was loaded per well of a 15% SDS–polyacrylamide gel electrophoresis gel and transferred onto polyvinylidene difluoride membrane. The membrane was blocked in 5% dry milk (Genesee Scientific, #20-241) or 0.1% casein (Sigma C5890-500G). Primary antibodies were used at 1:1,000 dilution, and secondary horseradish peroxidase antibodies were used at 1:5,000 dilution or secondary fluorescent antibodies were used at 1:15,000. Fluorescent secondary antibodies were visualized using a LI-COR Odyssey scanner. Quantification of band intensity was performed in ImageJ and all normalizations were to the shown loading control. For uncropped western blots, refer to Supplementary Fig. 17.

### Flow cytometry

Flow cytometry was performed on a BD FACS Calibur cytometer and analysed with BD CellQuest Pro.

*Propidium iodide viability staining*. A total of 100,000 cells were grown in a six-well TC dished with 3 ml media containing drug or control solution for 48 h. Media was collected, cells were washed with 1 ml PBS, which was pooled with the media, and then cells were trypsinized for 5 min in 1 ml Tryspin-EDTA. Trypsinized cells were then combined with supernatants, cells were centrifuged for 5 min at 500*g* and then resuspended in 400 μl iced PBS containing 1 μg ml^−1^ propidium iodide. Cells were then analysed on the flow cytometer.

*Acridine orange autophagosome staining*. A total of 100,000 cells were grown in a six-well TC dished with 3 ml media containing drug or control solution for indicated time points, staggered from the latest time point. Media was removed and adherent cells were stained by 1 μg ml^−1^ acridine orange for 15 min. Staining solution was aspirated, cells were washed once in 1 ml PBS, and then cells were trypsinized for 5 min in 1 ml Tryspin-EDTA. Trypsinized cells were then combined with 1 ml iced RPMI and centrifuged for 5 min at 500*g*. Supernatant was aspirated and cells were resuspended in 400 μl iced PBS. Cells were then analysed on the flow cytometer.

### Mouse models

All animal protocols were approved by the Institutional Animal Care and Use Committee (IACUC) of University of California: San Diego (UCSD), and all rules and regulations were followed during experimentation on animals. Experiments were powered to detect differences of 30% (http://homepage.divms.uiowa.edu/∼rlenth/Power/). No blinding was performed, since drug and control solutions were visually distinguishable. All mice were female, and COAST doses (250 mg kg^−1^ nelfinavir, 30 g kg^−1^ chloroquine, 2.24 mg kg^−1^ rapamycin, 150 mg kg^−1^ metformin and 4 mg kg^−1^ dasatinib, daily by gavage in 50% PEG400 in water) were determined using clinically safe doses as determined from a previous study^24^. All mice were included for the following experiments if above 18 g starting weight and with a healthy disposition before any injections. No mice were censored in these experiments.

In the subcutaneous model, 5 × 10^6^ OVCAR3 cells were injected into the right flank of 8–10-week-old female nude Nu/nu mice (*N*=7 per group). Mice were randomized when tumours were palpable. Treatment with control (gavage, daily, 50% PEG400) or COAST began when tumours reached 100 mm^3^, which was 14–20 days after cell injection. Mice were treated for 7 days and then killed 3 h following the last treatment. Tumours were removed and weighed as additional confirmation of the caliper size measurements.

For the chemo-resistant model, 5 × 10^6^ early passage LPPDOV cells were injected intraperitoneal (i.p.) into a female Nu/nu mouse, allowed to develop visible tumours, and ascites were collected and plated in complete RPMI on a TC-treated Petri dish. Non-adherent blood cells were washed off with RPMI, and then the adherent cells were trypsinized and transferred to a non-TC-treated plate for amplification. As soon as sufficient cells were grown to inject a cohort of mice (<5 passages), 3 million cells were injected i.p. into 8–10-week-old female Nu/nu mice. After injection, groups were normalized and randomized for mouse weight (*N*=10 for control group, *N*=7 for chemotherapy group and *N*=9 for COAST group). Ten days post cell injection, daily gavaging of COAST or control (50% PEG400) was performed for 15 days. In the cisplatin/docetaxel group, mice were additionally injected i.p. with 1 mg kg^−1^ cisplatin and 2.5 mg kg^−1^ docetaxel once per week starting at the first control treatment day for 2 weeks.

For the syngeneic OV model, 3 × 10^6^ mCherry labelled ID8-IP cells^27^, which have been passaged in the peritoneal cavity, were injected i.p. into syngeneic female C57BL/6 mice at 10 weeks of age. Mice of equal mean weights were used in each group (*N*=8 per group), randomized post-injection, and are the same cohort summarized in a previous study of ours^24^. Fourteen days after injection, one group received daily (seven times a week) vehicle gavage injections (50% PEG400), the C+N group received daily chloroquine and nelfinavir gavage (30 and 250 mg kg^−1^, respectively) and the COAST group received daily COAST gavage. Mice were monitored daily for distended abdomens following the first treatment injections. All mice were killed when ascites formation produced visible discomfort to control animals, which occurred after 14 days of treatment (28 days since cell injection). The peritoneum of the mice was exposed and any visible nodules on the peritoneum wall were surgically dissected along with the liver and ovaries. These tissues were then imaged with the OV100 Small Animal Imaging System (Olympus). Bright-field, GFP and mCherry channel information were collected and only red fluorescent (but not green autofluorescent) punctae area was quantified in ImageJ. Fluorescent area was mathematically converted into tumour volume assuming spherical shape of the tumour and circular shape of the fluorescent area. Any bloody ascites present upon initial opening of the peritoneum was transferred by P1000 micropipette into a 15 ml conical tube and volume determined by micropipette. In the longer-term safety experiment, the experiment was performed identically, except mice were treated by COAST for a period of 8 weeks with five daily doses (daily excluding weekends).

### Data availability

All the data that support the findings of this study are available within the article and Supplementary Files, or available from the authors upon request.

## Additional information

**How to cite this article:** Delaney, J. R. *et al*. Haploinsufficiency networks identify targetable patterns of allelic deficiency in low mutation ovarian cancer. *Nat. Commun.* **8**, 14423 doi: 10.1038/ncomms14423 (2017).

**Publisher's note:** Springer Nature remains neutral with regard to jurisdictional claims in published maps and institutional affiliations.

## Supplementary Material

Supplementary Information Supplementary Figures and Supplementary Tables

Supplementary Data 1 Output of the significantly altered HAPTRIG-KEGG pathways and impactful genes in the TCGA OV cohort. KEGG pathways and corresponding p value scores for HAPTRIG analysis are compared, with the most impactful 5 genes influencing pathway score in the significantly altered direction.

Supplementary Data 2 Output of the significantly altered HAPTRIG-KEGG pathways and impactful genes in the 2009 serous OV cohort. KEGG pathways and corresponding p value scores for HAPTRIG analysis are compared, with the most impactful 5 genes influencing pathway score in the significantly altered direction.

Supplementary Data 3 Output of the significantly altered HAPTRIG-KEGG pathways and impactful genes in the 2009 endometrioid OV cohort. KEGG pathways and corresponding p value scores for HAPTRIG analysis are compared, with the most impactful 5 genes influencing pathway score in the significantly altered direction

Supplementary Data 4 Output of the MsigDB Hallmark pathways as tested by HAPTRIG. Hallmark pathways and corresponding p value scores for HAPTRIG analysis are compared, with the most impactful 5 genes influencing pathway score in the significantly altered direction. The TCGA OV cohort was used

Supplementary Data 5 Packet of tables as examples to be used with supplied HAPTRIG code (Supplemental Software 1). Note that OV gistic data is cut to first 100 patients to allow for journal data limit requirements.

Supplementary Software 1 HAPTRIG R program. Open source code is provided to run HAPTRIG using R software
