# Supplementary material for: Haploinsufficiency networks identify targetable patterns of allelic deficiency in low mutation ovarian cancer
Source: Nat Commun. 2017 Feb 15;8:14423. doi: 10.1038/ncomms14423 (PMC5316854; doi:10.1038/ncomms14423)
Supplement: Supplementary Information — Supplementary Figures and Supplementary Tables [file ncomms14423-s1.pdf]

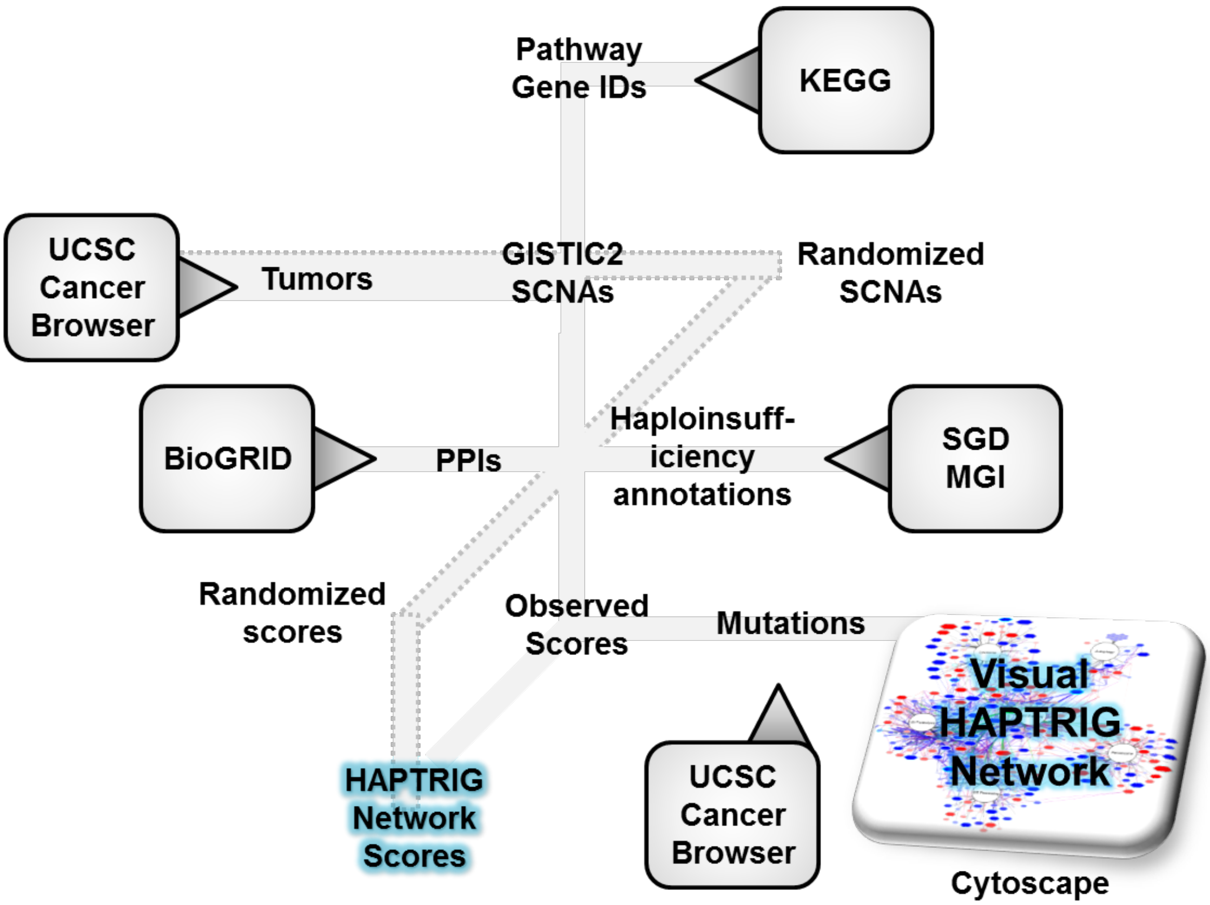

Supplementary Figure 1. HAPTRIG network scoring workflow. Data and annotation sources are indicated by filled boxes. Outputs from sources are indicated by overlaid text. Calculations can be found in Methods or Supplemental Software 1.

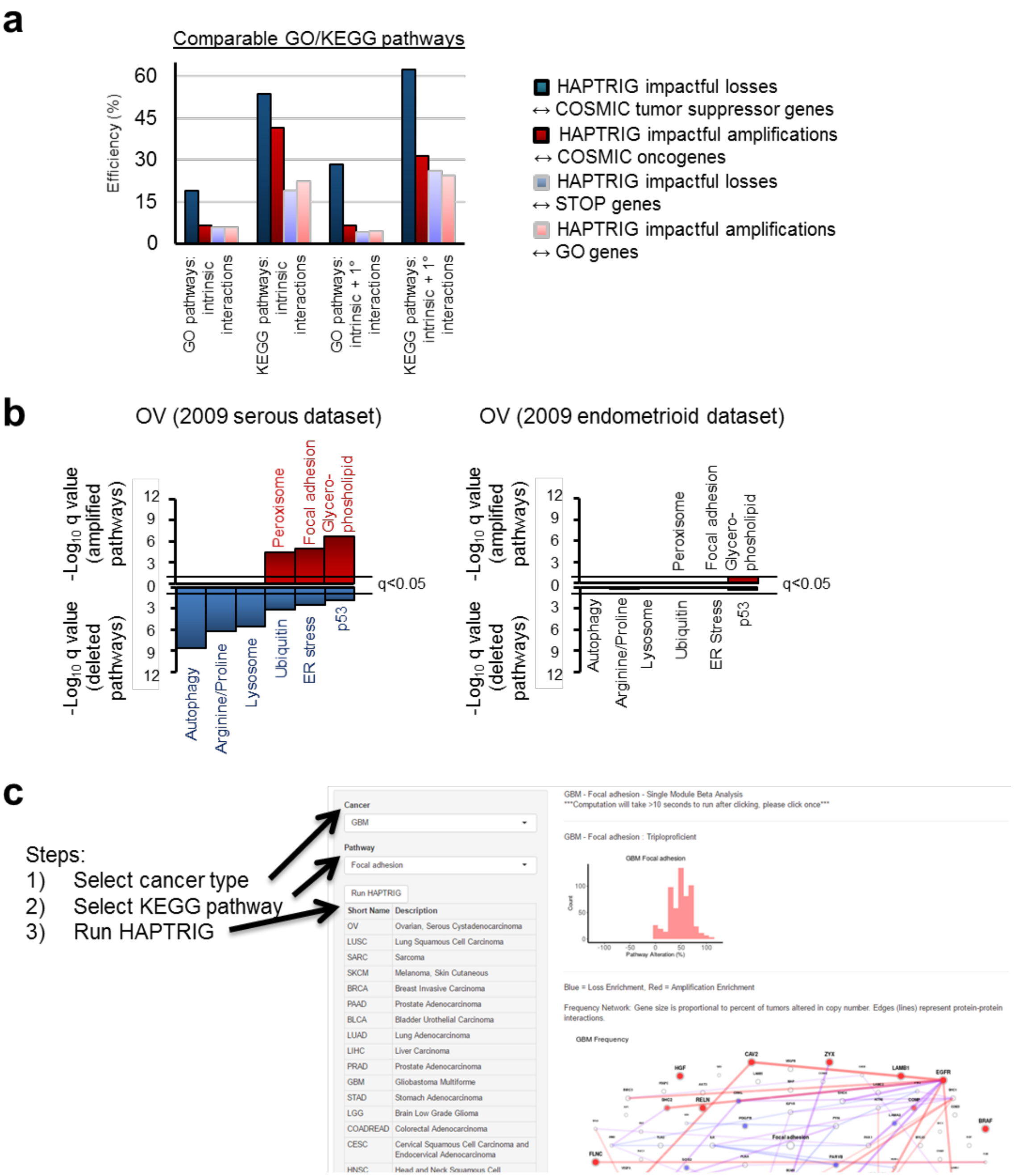

Supplementary Figure 2. Additional HAPTRIG controls and datasets. (a) Comparable GO/KEGG pathways (see Supplementary Data 4. Includes proteostasis pathways and 11 other tumor-related pathways) were scored by HAPTRIG, either using only protein-protein interactions within a pathway (intrinsic) or with protein-protein interactions which could include a gene outside the pathway (1° interactions). Unlike the 187-pathway analysis, 1° interactions better prioritized KEGG tumor suppressor genes and thus were used in the proteostasis network scores for Fig. 3. (b) Additional, independent OV datasets were used to verify HAPTRIG findings. As expected, proteostasis pathways were downregulated in the serous dataset, but not the endometrioid dataset. Using all 187 pathways, autophagy was the #2 suppressed pathway in the serous dataset (Supplementary Data 2), but did not reach significance in the endometrioid dataset (Supplementary Data 3). (c) Screenshot from the free, available web tool to perform systematic HAPTRIG analysis on 21 cancer types and 187 KEGG pathways.

The tool is available at: https://delaney.shinyapps.io/HAPTRIG_Single_Module_Beta/

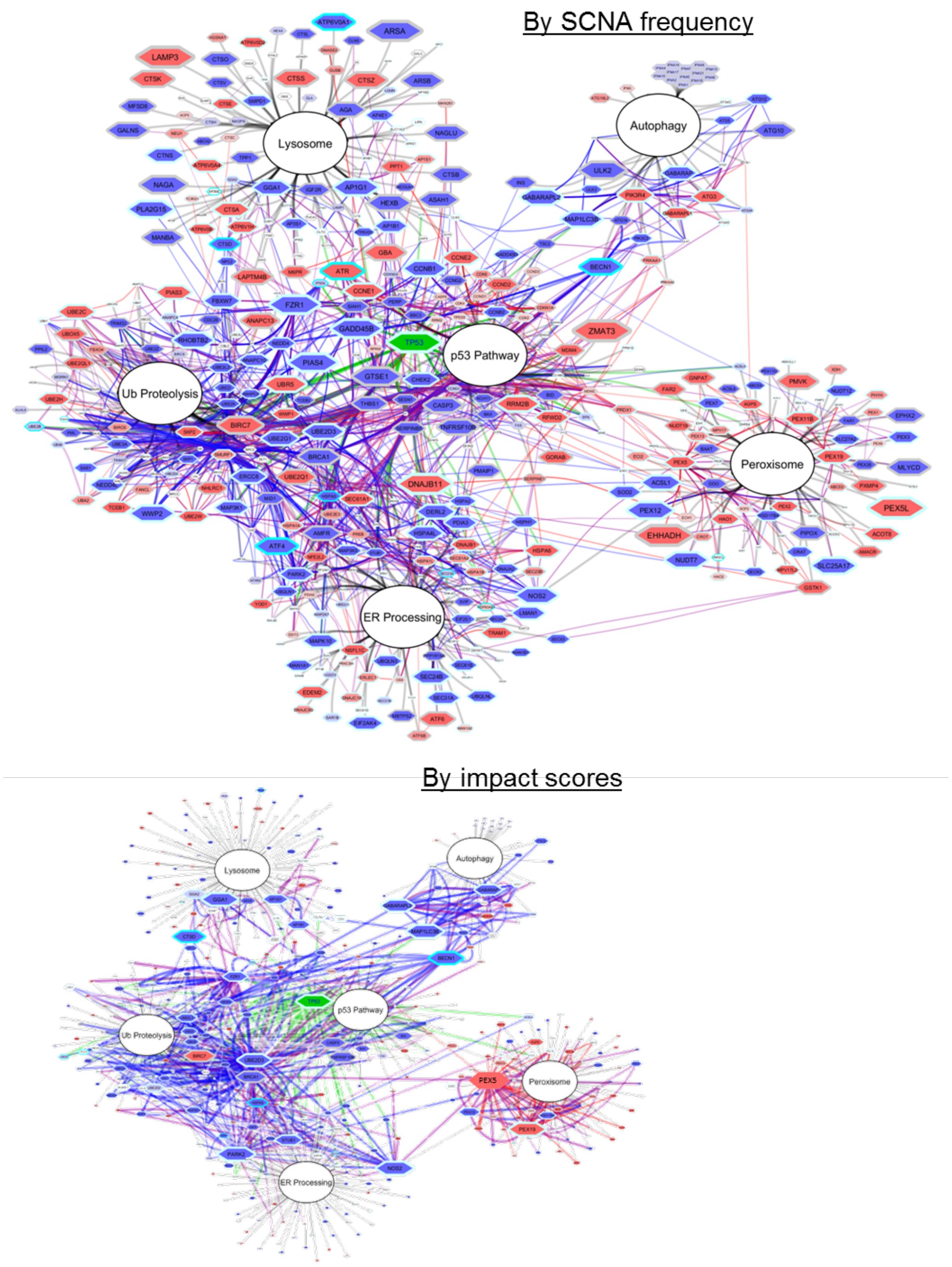

Supplementary Figure 3. Visual HAPTRIG networks for OV. Gene nodes and edge protein-protein interactions are displayed with size proportionate to the prevalence of the gene change within the cancer type (top panel) or by the level of the gene's impact on pathways as scored by HAPTRIG (bottom panel). Edges are only drawn if one gene is affected by copy number changes in >33% of patient tumors (top panel) or if the impact of one gene in the edge is 1 z-score away from the mean impact (bottom panel). A red color is assigned if the majority of copy number changes are positive, and blue if they are negative. Green fill and edges indicate genes mutated in >10% of the tumor cohort. Node outlines are highlighted in cyan if haploinsufficiency annotations are associated with that gene: bright cyan if murine evidence exists and light cyan if yeast homologue evidence was found. Grey edges connect genes to corresponding KEGG pathways. *IFNA* genes are visually clustered due to their locally arrayed position on chromosome 9p21.3.

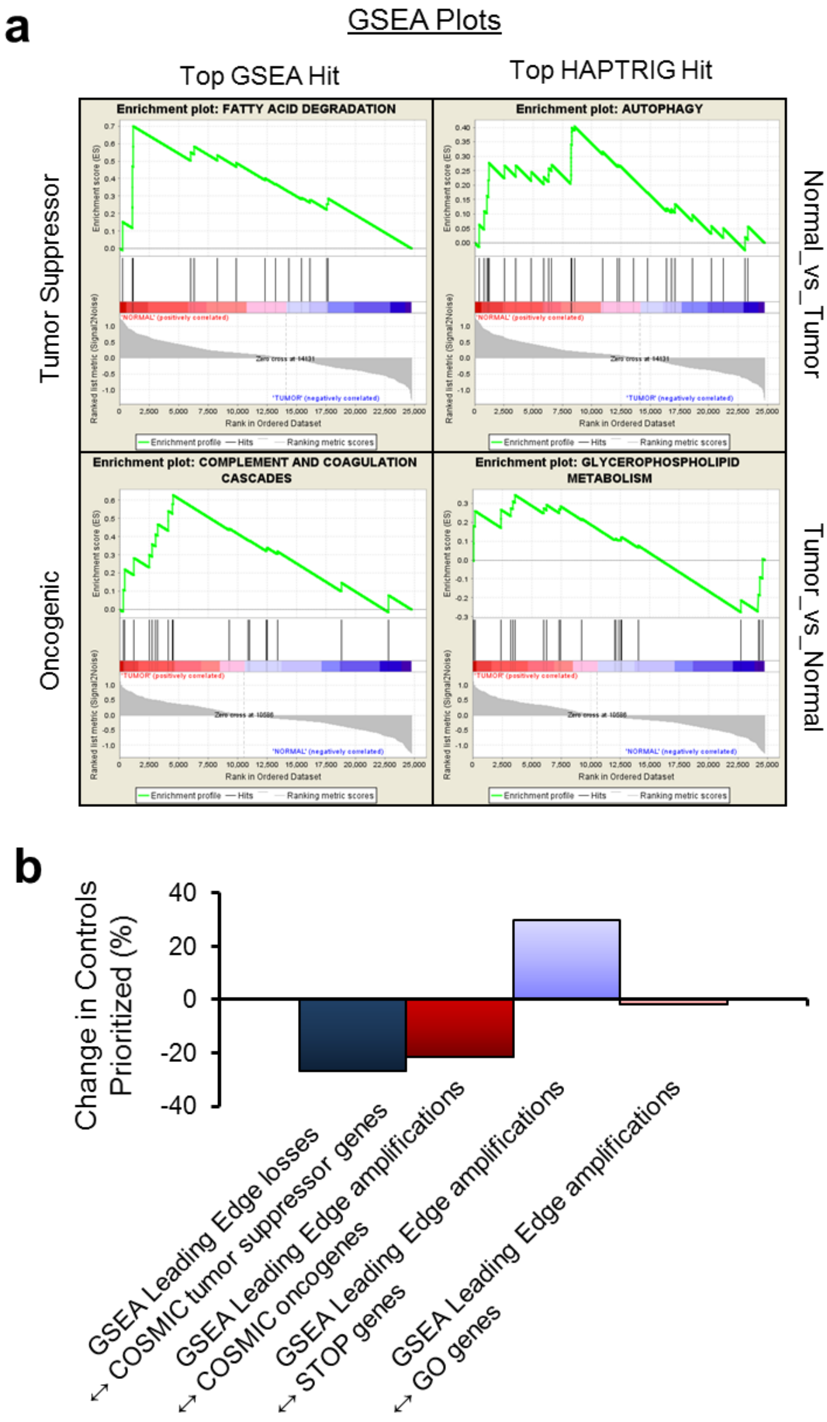

Supplementary Figure 4. Comparison of HAPTRIG to GSEA. (a) Copy-number data for OV was input into GSEA using identical pathways as HAPTRIG. Results of GSEA for top-hit tumor suppressors and oncogenic pathways are shown for both GSEA and HAPTRIG. All pathways and direct comparisons are enumerated in Supplemental Data 8. (b) Benchmarking of GSEA in comparison to HAPTRIG was performed as in Figure 1. HAPTRIG identified more tumor suppressor genes and oncogenes as most impactful compared to leading edge analysis of GSEA, but the opposite was observed for prioritization of STOP genes, suggesting the methods are complementary.

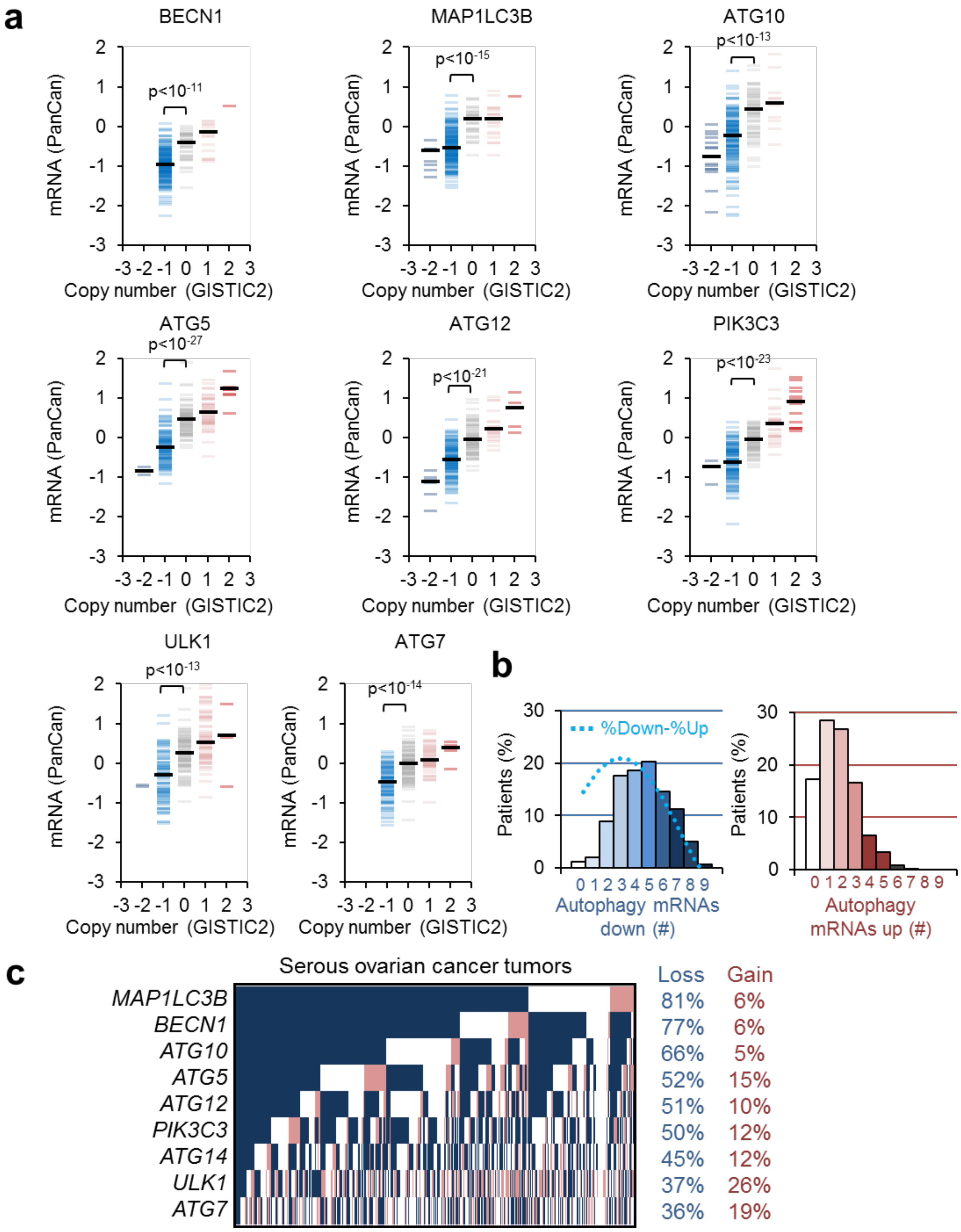

Supplementary Figure 5. Monoallelic losses of autophagy genes match mRNA suppression. (a) GISTIC2 calls of core autophagy genes are compared to microarray RNA expression levels within OV. Microarray mRNA comparisons are displayed since there are a higher number of samples than RNAseq. (b) Per patient data on number of autophagy mRNA displaying downregulation versus upregulation. Z score and EXP score cutoffs were set at +/-0.5. Autophagy gene queries match the canonical core initiation genes shown in (a). (c) TCGA OV tumors are compared for coincident losses of autophagy genes: each column represents a single tumor. Blue corresponds to allelic deletion, red to allelic gain.

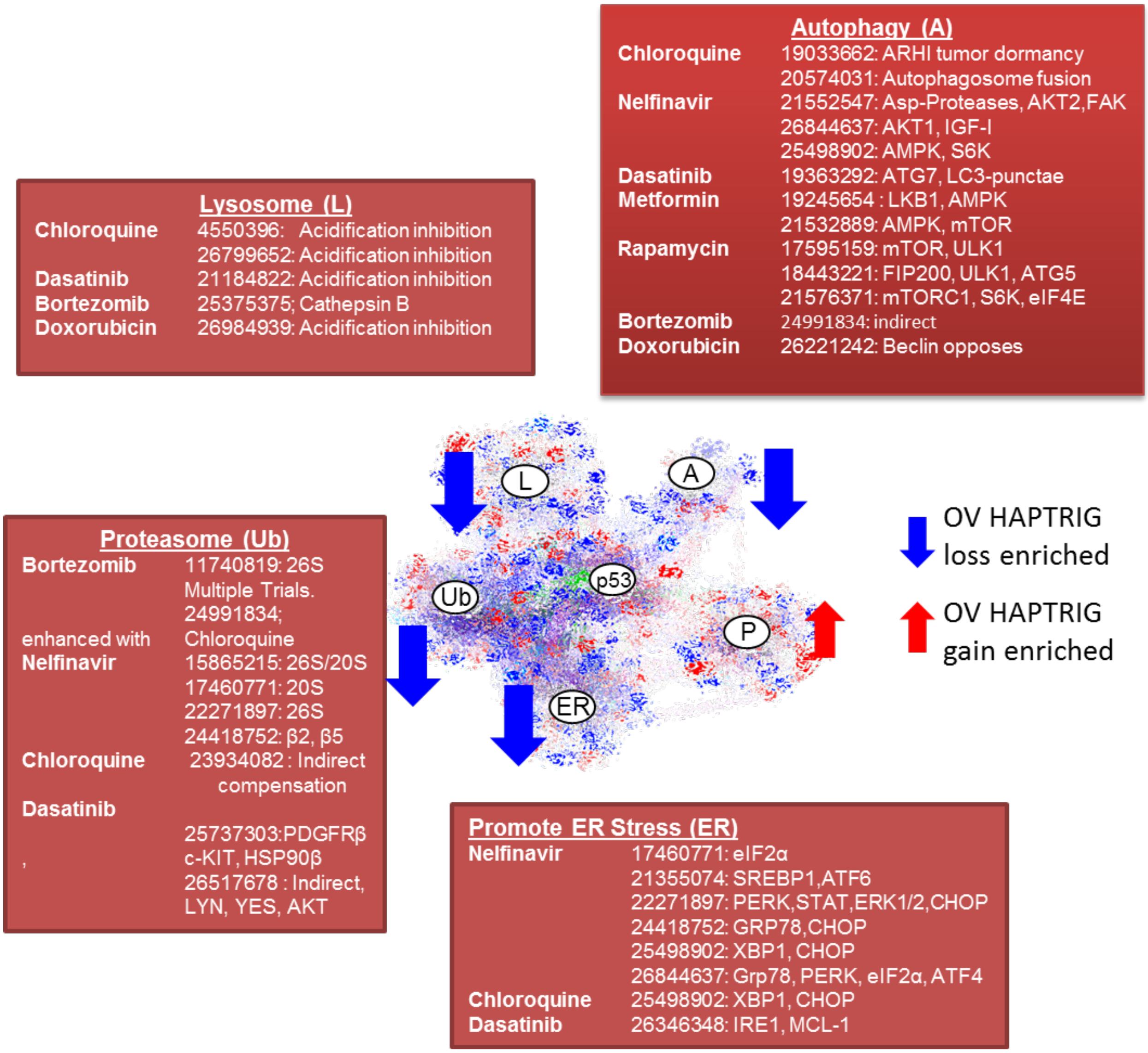

Supplementary Figure 6. Approved drugs targeting the monoallelically disrupted proteostasis pathways of OV. Summary of altered proteostasis pathways in OV and FDA-approved drugs impacting the pathway, with corresponding publication report (PMID number shown).

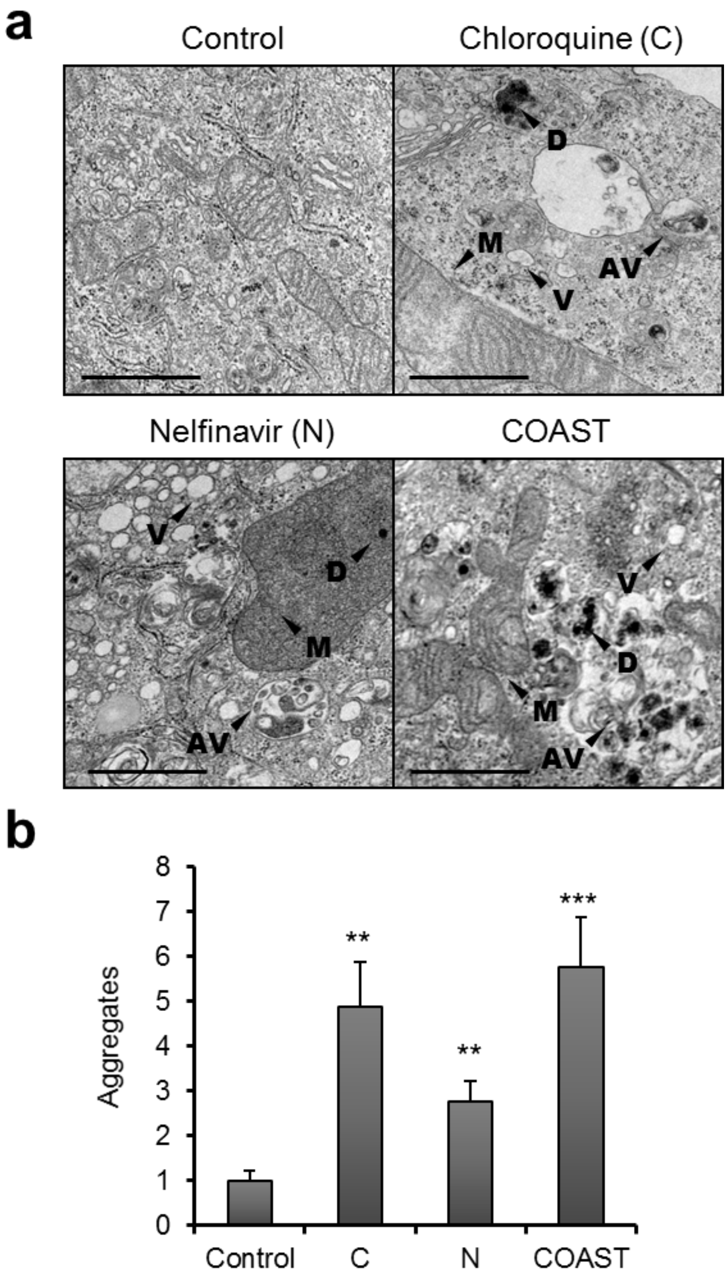

Supplementary Figure 7. Quantitation of protein aggregation by electron microscopy. (a) Transmission electron micrographs of OVCAR3 cells treated for 12 hours with COAST components or the full combination (metformin 10μM, chloroquine 10μM, nelfinavir 10μM, rapamycin 10nM, and/or dasatinib, 50nM). Labels: M, Mitochondria, V, Vesicle, AV, Autophagic Vesicle, D, Dense protein aggregate. Scale bar is 1μm. (b) Images were blinded and then quantified for electron dense protein aggregates. Data represent mean ± s.e.m. from a single experiment. Aggregate quantitation is relative to control cells. $^{**}p<0.01$, $^{***}p<0.001$ by two-tailed student's t-test.

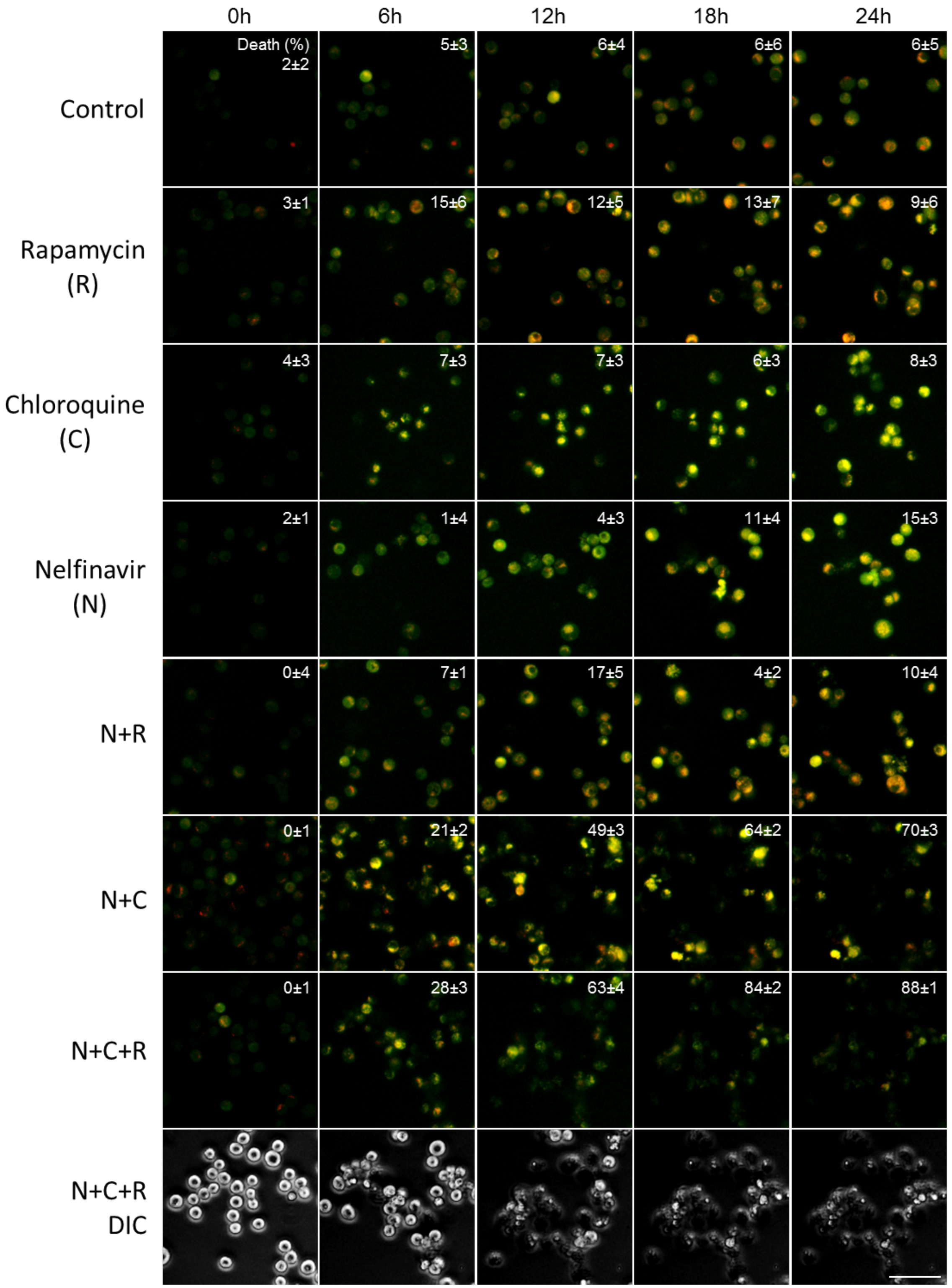

0h
6h
12h
18h
24h
Control
Rapamycin (R)
Chloroquine (C)
Nelfinavir (N)
N+R
N+C
N+C+R
N+C+R DIC
Death (%)
2±2
5±3
6±4
6±6
6±5
3±1
15±6
12±5
13±7
9±6
4±3
7±3
7±3
6±3
8±3
2±1
1±4
4±3
11±4
15±3
0±4
7±1
17±5
4±2
10±4
0±1
21±2
49±3
64±2
70±3
0±1
28±3
63±4
84±2
88±1

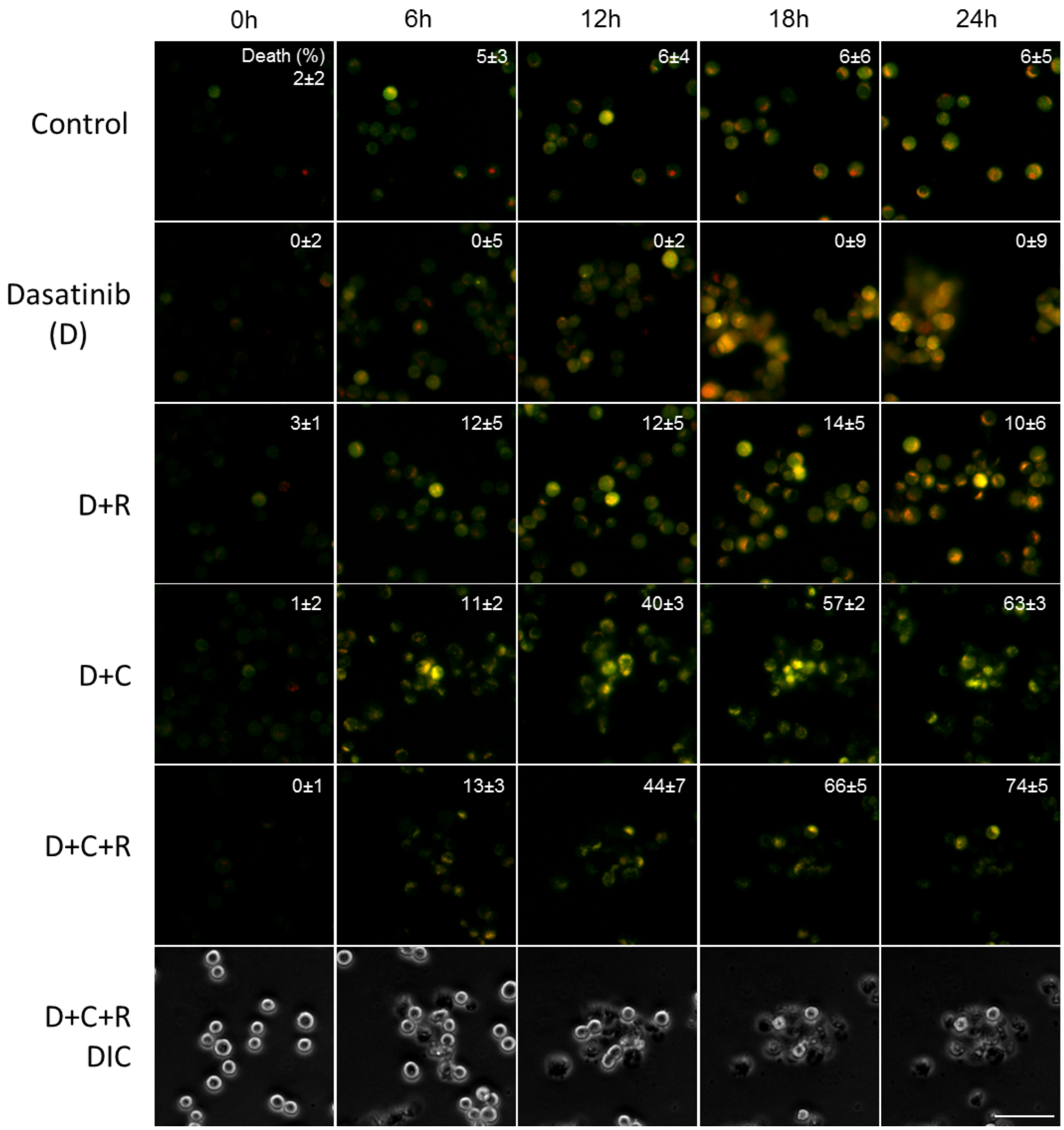

Supplementary Figure 8. **Live autophagic flux microscopy of COAST on OVCAR3 cells.** OVCAR3 cells with a virally integrated mCherry-GFP-LC3 construct were studied for autophagic flux by live microscopy. Autophagolysosome formation quenches the GFP signal due to acidification, which mCherry is resistant to and remains fluorescing red. Yellow punctae thus primarily represent autophagosome structures which have not passed the lysosome fusion stage. Cells were treated with drugs (COAST component drugs which included chloroquine (C, 10μM), nelfinavir (N, 10μM), rapamycin (R, 100nM), and/or dasatinib (D, 100nM)) and then imaged live in normal cell culture conditions by spinning disc microscopy. Lysed cell percentages are shown by inset text. Three independent experiments were performed with representative images shown, numbers represent mean ± s.e.m. Scale bar is 40μm.

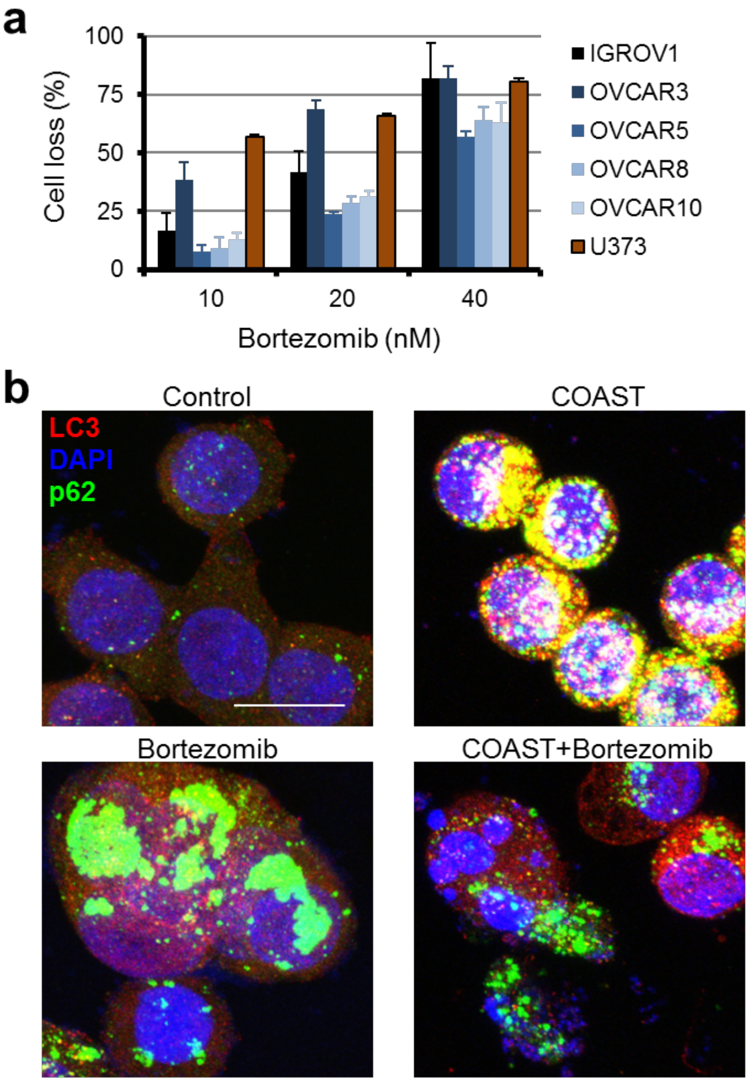

c

Most frequent adverse event

Drug	G1-2	G3	G4	Safety reference
Chloroquine	2%	-	-	PMID 10759574
Nelfinavir	17%	-	-	FDA (NDA 21-503)
Rapamycin	35%	15%	2%	PMID 21752435
Dasatinib	17%	5%		FDA (NDA 21-986)
Metformin	8-13%	-	-	PMID 17638715
**Bortezomib**	**100%**	**61%**	**14%**	**FDA (NDA 21-602)**

Supplementary Figure 9. Bortezomib is cytotoxic and induces aggregates, but at the cost of clinical safety. (a) Crystal violet viability assay of cells treated with indicated concentrations of bortezomib for 48 hours. (b) Immunofluorescence of OVCAR3 cells treated with bortezomib (40nM) and/or COAST (the combination of metformin, chloroquine, nelfinavir, rapamycin, and dasatinib at 10μM, 10μM,10μM, 10nM, and 50nM, respectively) for 24 hours. Scale bar is 20μm. (c) Safety data from clinical trials suggest non-overlapping toxicity and low toxicity for all COAST drugs used here, with the exception of bortezomib. Due to the high expectation of toxicity with bortezomib, especially in combination, we did not include the drug in further analyses.

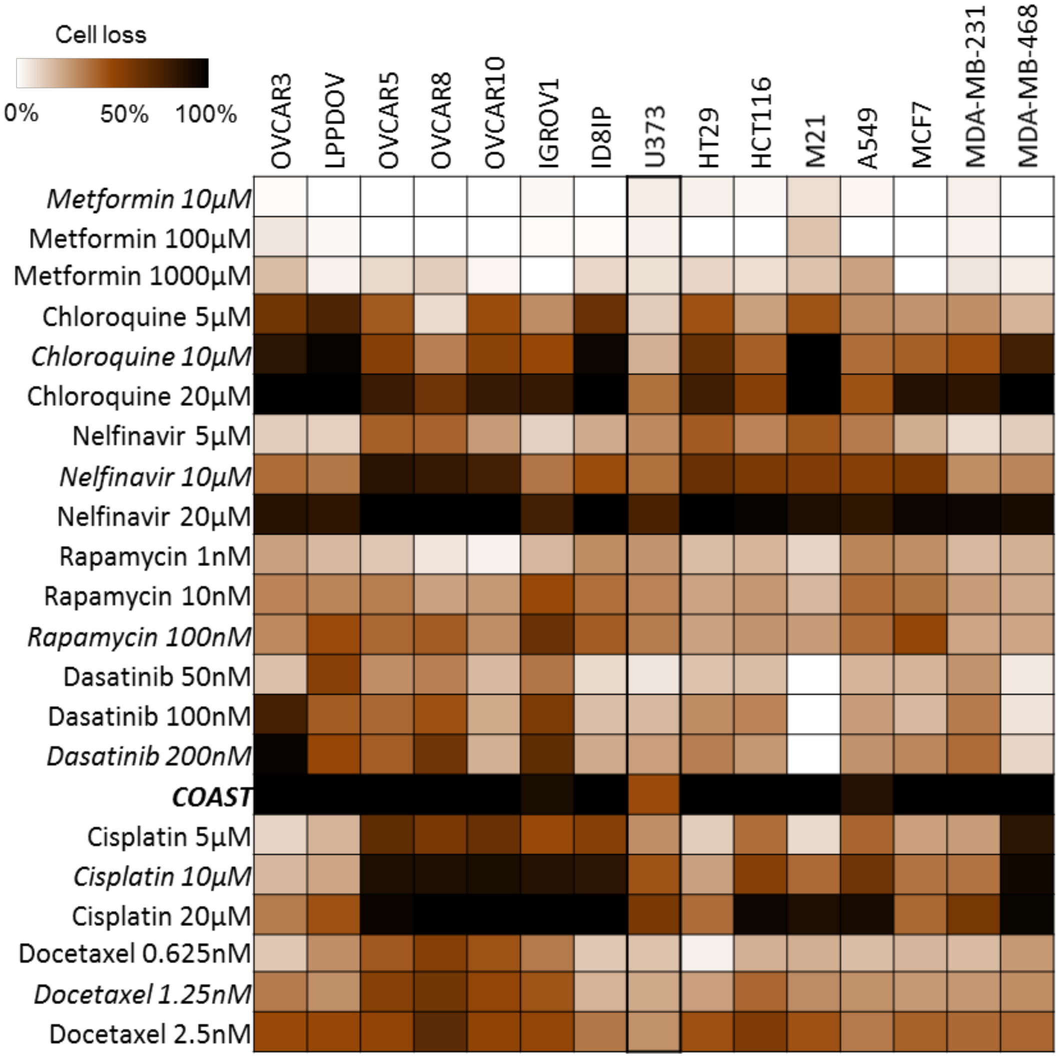

Supplementary Figure 10. Ovarian cancer cell lines are sensitive to autophagic drugs at physiological levels. Five human ovarian cancer cell lines (OVs and IGROV1) (note that IGROV1 is unlikely to be serous OV), one low passage patient-derived ovarian cancer (LPPDOV), a mouse ovarian cancer (ID8-IP) and other cancer types are tested for their response to metformin (M), chloroquine (C), nelfinavir (N), rapamycin (R), and dasatinib (D) as well as the common chemotherapeutics cisplatin and docetaxel for reference. Control U373 glioblastoma cells exhibit much less proteotoxic death than any other cancer line. Other cancer types: colorectal (HT29, HCT116), melanoma (M21), lung (A549), and breast (MCF7, MDA-MB-231). Growth is measured by a 48 hour crystal violet assay. Peak blood concentration of drug is indicated in italics. Here, the combination of M,C,N,R, and D at 10μM, 10μM, 10μM, 10nM, and 50nM, is used as COAST, respectively. Mean data from three independent experiments are shown.

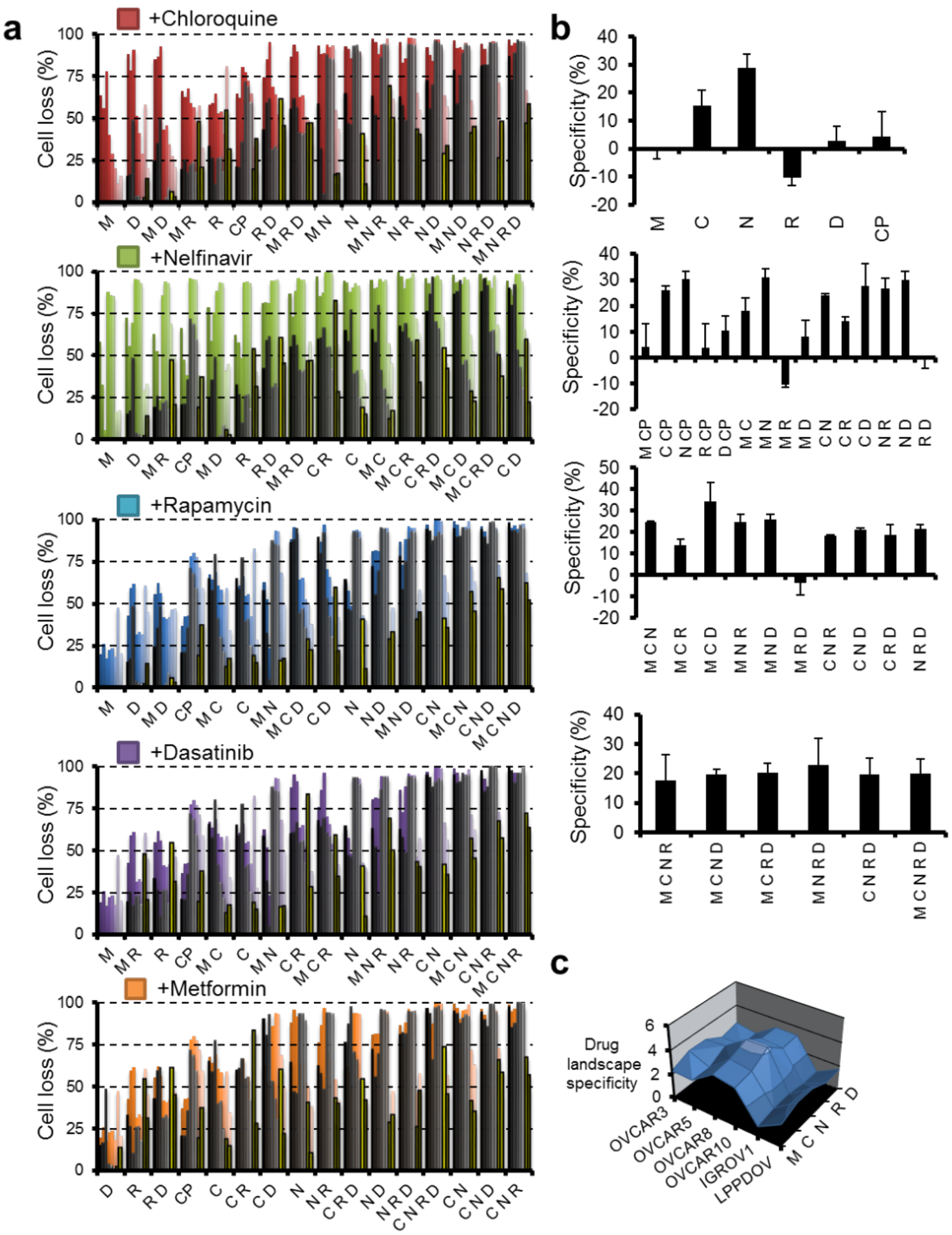

Supplementary Figure 11. Fully combinatorial autophagy drug studies indicate COAST is more effective than single drugs. (a) Crystal violet proliferation assays were performed in OVCAR3,5,8,10 cells (grey bars, in order) and autophagy competent (see Supplementary Figure 14) IGROV1 and U373 cells (yellow bars, in order). Each panel shows N-1 drug combinations on the x-axis, with the added drug growth inhibition indicated by the color bars superimposed behind the growth inhibition of the drug indicated on the x-axis. Higher colored bars indicate additivity of the tested drug as compared to a lower order combination. CP indicates cisplatin, at 10μM. Other drugs are at standard COAST concentrations (metformin, M, 10μM, chloroquine, C, 10μM, nelfinavir, N, 10μM, rapamycin, R, 10nM, and/or dasatinib, D, 50nM). Data are from eleven independent experiments, with mean values shown. (b) Specificity of drugs and combinations determined from the data in (a); the average of autophagy competent (U373 and IGROV1) lines' growth inhibition is subtracted from the average of autophagy deficient lines' growth inhibition. Mean values ± s.e.m. shown. (c) Drug landscape specificity indicates Log_2 percent increases in cell loss averaged across 17 drug combinations containing the indicated drug when compared to *de-facto* resistant cell line U373. All OVs tested with the exception of IGROV1 show increased sensitivity to COAST drug combinations.

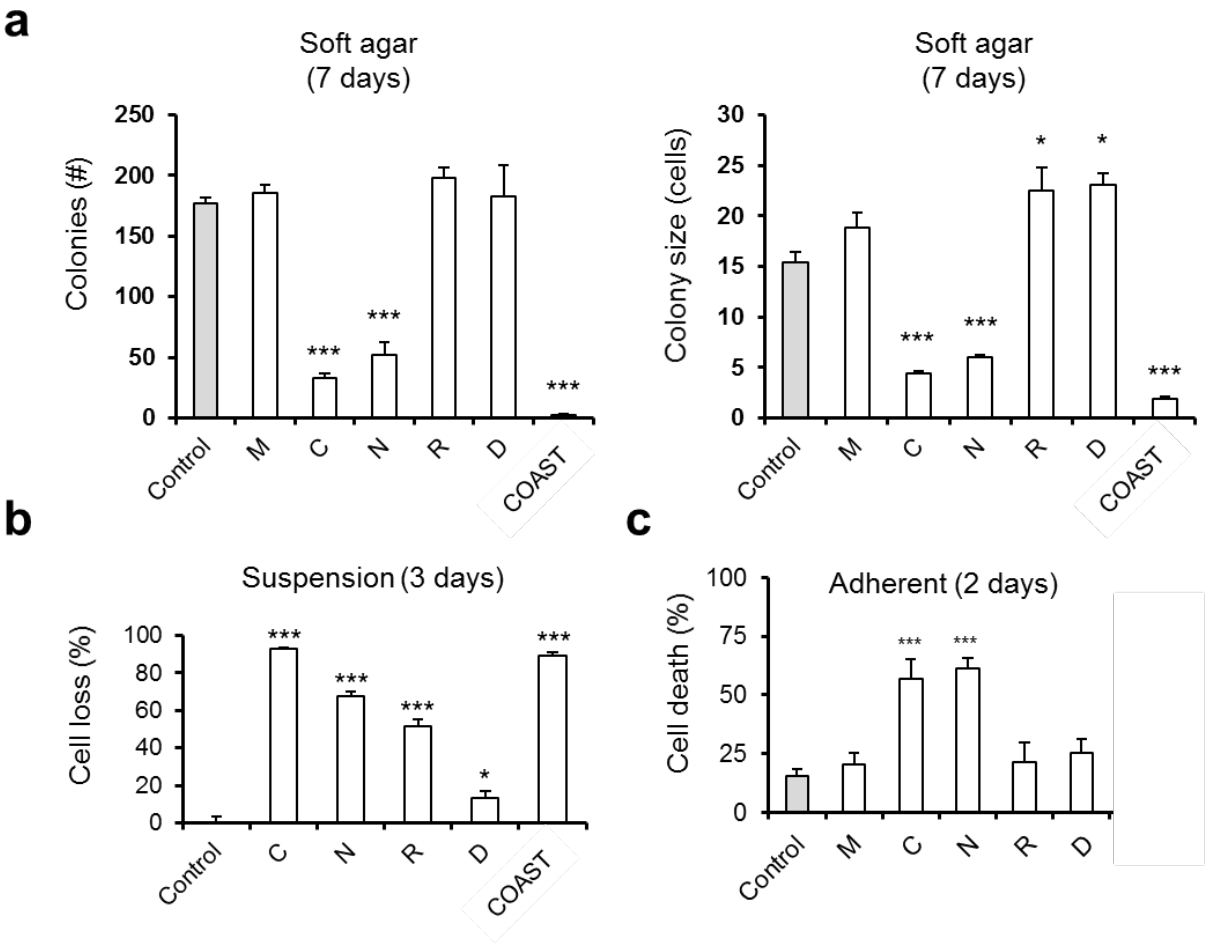

Supplementary Figure 12. Cytotoxicity of COAST drugs in colony formation and suspension. (a) OVCAR3 cells were embedded in soft agar and allowed to proliferate for 7 days following treatment with the indicated drugs (COAST component drugs which included metformin (M, 10μM), chloroquine (C, 10μM), nelfinavir (N, 10μM), rapamycin (R, 10nM), and/or dasatinib (D, 50nM), COAST is the five drug combination). Experiment was performed three times with a representative mean±s.e.m. shown, *p<0.05, ***p<0.001 by two-tailed student's t-test. (b) OVCAR3 cells were grown in polyHEMA plates to force cells into a non-adherent state and tested for drug sensitivity as in (a) for 72 hours of growth. Trypan blue was used to measure cell viability and a ViCell cell counter was used to measure cell counts. Experiment was performed three times with combined mean±s.e.m. shown, *p<0.05, ***p<0.001 by two-tailed student's t-test. (c) PI staining as a proxy of cell death of adherently grown OVCAR3 cells treated with the indicated drugs for 48 hours, as measured by flow cytometry. Experiment was performed three times with combined mean±s.e.m. shown, ***p<0.001 by two-tailed student's t-test.

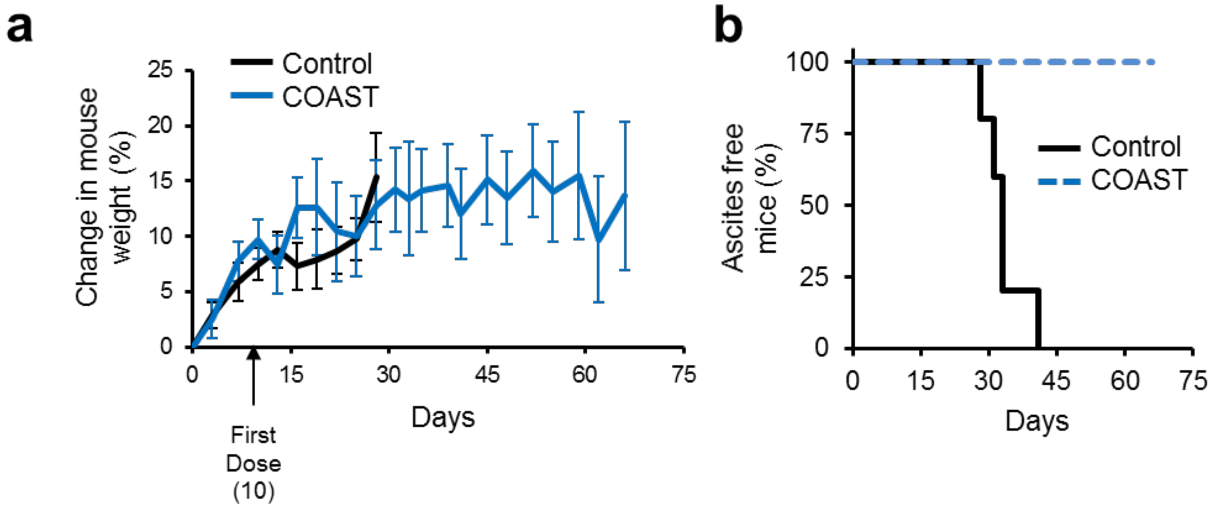

Supplementary Figure 13. Longer term COAST therapy does not yield weight loss systemic toxicity. C57BL/6 mice were inoculated IP with ID8-IP tumors and then treated by gavage with COAST identically as in Fig. 4, except for 8 weeks rather than 2 weeks. The mice never lost weight during the course of therapy (a), even long after all control mice were euthanized due to ascites-induced morbidity (b).

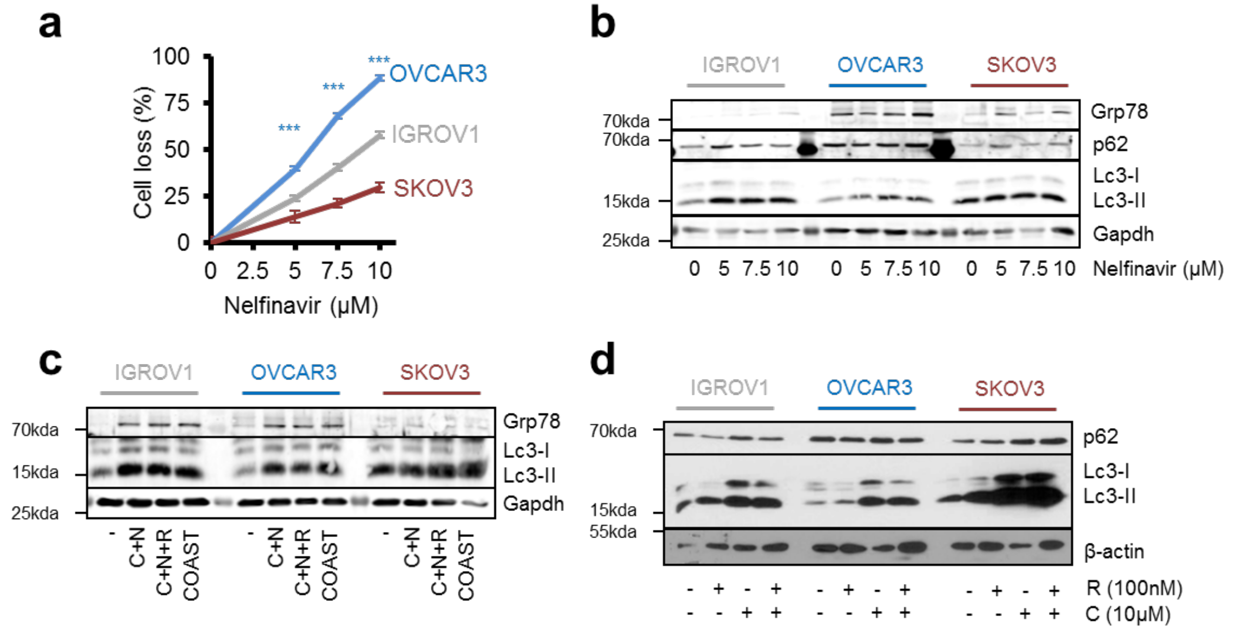

Supplementary Figure 14. Inadequate autophagy in OVCAR3 following nelfinavir drug treatment. (a) Crystal violet viability assay after 48 hours of nelfinavir treatment. Data represent the mean ± s.e.m. from eight independent experiments. ***p<0.001 by two-tailed student's t-test, comparing OVCAR3 to either IGROV1 or SKOV3 (all are ***). (b-d) Western blots of autophagosomal Lc3-II indicate reduced accumulation of autophagosomes in OVCAR3 and increased levels of ER-stress marker Grp78 when treated with nelfinavir (b), combinations of drugs (c) including chloroquine (10μM, C), nelfinavir (10μM, N), rapamycin (R, 10nM), or more (COAST, which additionally includes metformin 10μM and dasatinib 50nM), or with combinations of the autophagy activator rapamycin with the autophagy clearance inhibitor chloroquine (d). Lysates were generated 24h after initiation of treatment. Lysates from three independent experiments were analyzed and a representative blot is shown.

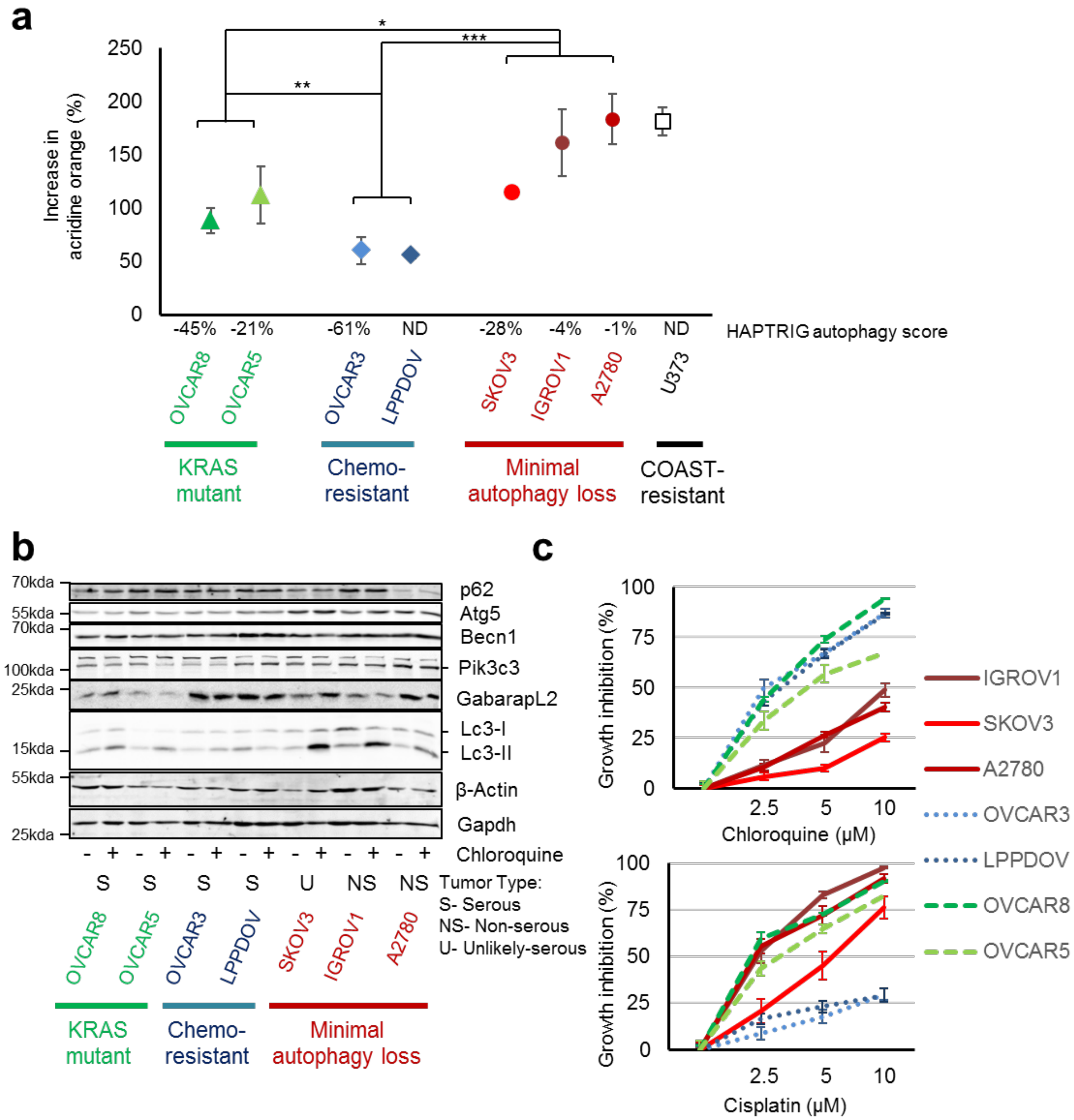

Supplementary Figure 15. Additional OV cell lines tested for autophagy. Related to Fig. 4. (a) OVCAR3 and LPPDOV have delayed accumulation of acidic vacuoles including autophagosomes and lysosomes, as measured by acridine orange flow cytometry, when treated with the autophagy/lysosome inhibitor chloroquine (10μM, 24h). *KRAS* mutant but autophagy gene-deleted OVCAR8 and OVCAR5 have somewhat less of a delay, compared to cell lines with less core autophagy gene deletions: SKOV3, IGROV1, and A2780. HAPTRIG autophagy pathway scores are shown for comparison, when data are available (if not, then "ND"). Data represent the mean ± s.e.m. from three independent experiments. (b) Western blots of autophagosomal Lc3-II and other autophagy genes for the cell lines tested throughout this paper. Lysates from three independent experiments were analyzed and a representative blot is shown. Chloroquine treatment was 24h. (c) Crystal violet 48h cell death assays are shown for all cell lines for comparison. Of note, cisplatin resistance is not associated with chloroquine resistance.

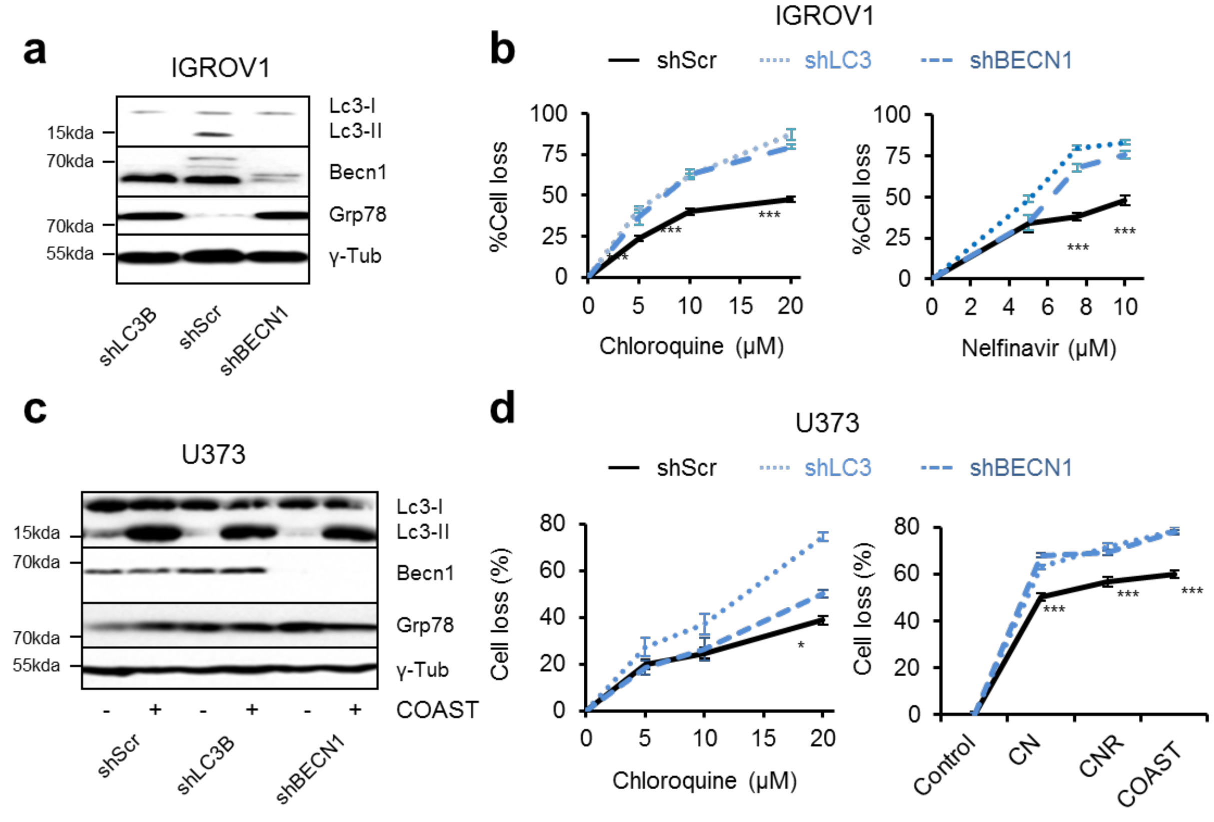

Supplementary Figure 16. LC3 and BECN1 gene suppression sensitizes IGROV1 and U373 cells to COAST. (a) IGROV1, a non-serous and autophagy competent ovarian cancer, was stably knocked down for the genes *LC3* and *BECN1*. Moderate knockdown clones (n=2) were selected for subsequent tests as a haploinsufficient mimetic. Lysates from three independent experiments were analyzed and a representative blot is shown. (b) Proliferation assays of IGROV1 knockdown cells in the presence of the COAST drugs chloroquine or nelfinavir. Data represent the mean ± s.e.m. from five independent experiments. ***$p<0.001$ by two-tailed student's t-test, indicating shScr is less sensitive than both shBECN1 and shLC3. (c) U373, a cell line found to be resistant to COAST drugs, was stably knocked down for the genes *LC3* and *BECN1*. Lysates from three independent experiments were analyzed and a representative blot is shown. (d) Proliferation assays of U373 knockdown cells in the presence chloroquine of COAST drug combinations (C, Chloroquine 10μM, N, Nelfinavir 10μM, R, Rapamycin 10nM, COAST is CNR plus 10μM metformin and 50nM dasatinib). Data represent the mean ± s.e.m. from three independent experiments. *$p<0.05$, ***$p<0.001$ by two-tailed student's t-test, indicating shScr is less sensitive than both shBECN1 and shLC3.

Fig. 4d

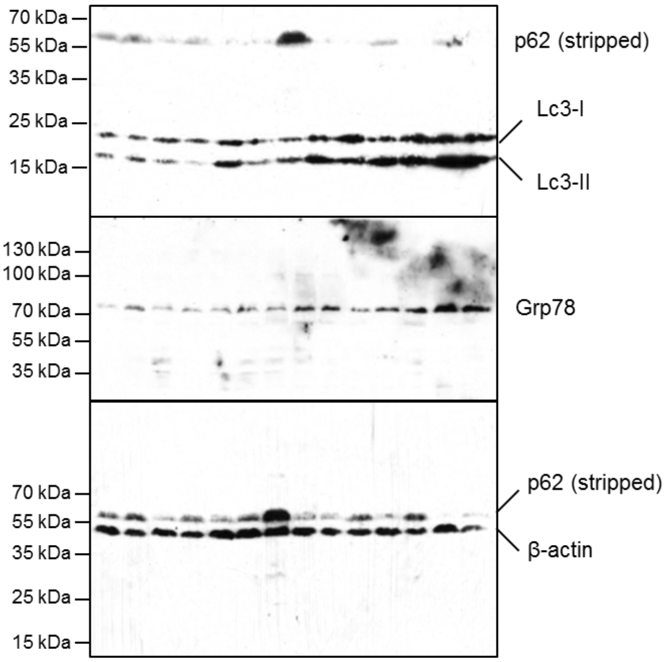

Fig. 5d

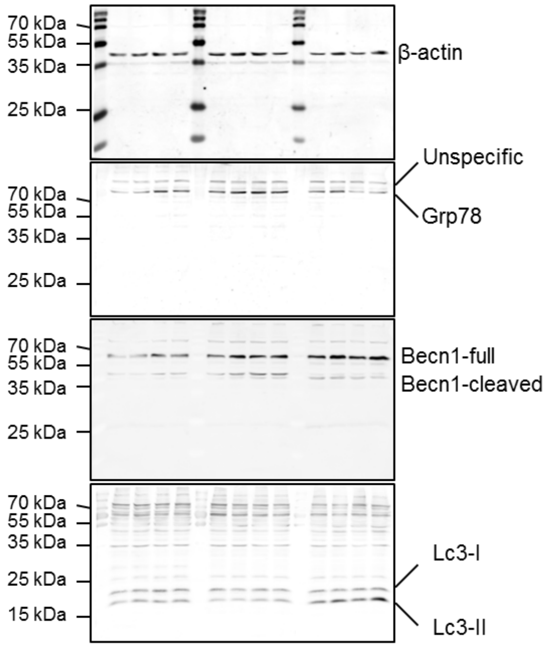

Fig. 5f

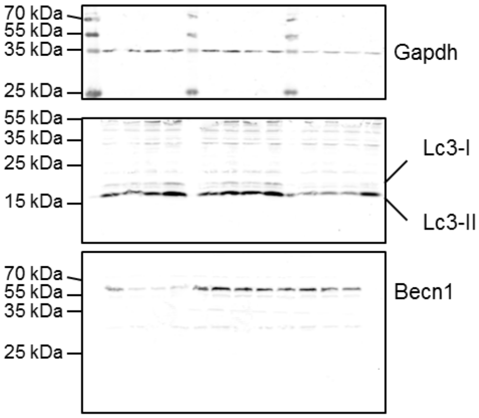

Supplementary Figure 17. Uncropped western blots.

Pathway	HAPTRIG Rank (-)	HAPTRIG Rank (+)	HAPTRIG Result	HAPTRIG p value	HAPTRIG q value	GSEA Rank (-)	GSEA Rank (+)	Rank diff-erence	GSEA NES	GSEA p-val	GSEA q-val
Autophagy	1		Haploinsufficient	6.13E-73	1.15E-70	10		-9	-1.45	ns	ns
FoxO signaling pathway	2		Haploinsufficient	4.5E-70	8.42E-68	15		-13	-1.33	ns	ns
Arginine and proline metabolism	3		Haploinsufficient	8.57E-70	1.60E-67	11		-8	-1.41	ns	ns
Adipocytokine signaling pathway	4		Haploinsufficient	2.45E-66	4.58E-64	2		2	-1.88	0.002	ns
Notch signaling pathway	5		Haploinsufficient	1.33E-63	2.49E-61	54		-49	-1.00	ns	ns
Longevity regulating pathway	6		Haploinsufficient	1.74E-48	3.25E-46	28		-22	-1.16	ns	ns
Fatty acid degradation	7		Haploinsufficient	1.05E-44	1.96E-42	1		6	-2.05	0	0.004
AMPK signaling pathway	8		Haploinsufficient	1.36E-43	2.54E-41	19		-11	-1.27	ns	ns
Ubiquitin mediated proteolysis	9		Haploinsufficient	4.48E-41	8.38E-39	42		-33	-1.07	ns	ns
Neuroactive ligand receptor interaction	10		Haploinsufficient	5.49E-39	1.03E-36	22		-12	-1.26	ns	ns
Retinol metabolism	11		Haploinsufficient	5.69E-39	1.06E-36	6		5	-1.62	0.031	ns
Huntington disease	12		Haploinsufficient	1.83E-36	3.42E-34	23		-11	-1.23	ns	ns
Glycolysis Gluconeogenesis	13		Haploinsufficient	2.1E-36	3.93E-34	3		10	-1.73	0.01	ns
HIF1 signaling pathway	14		Haploinsufficient	9.13E-35	1.71E-32	9		5	-1.54	0.019	ns
Lysosome	15		Haploinsufficient	7.46E-34	1.40E-31	13		2	-1.38	0.05	ns
Non small cell lung cancer	16		Haploinsufficient	7.34E-33	1.37E-30		38	NA	0.96	ns	ns
Thyroid hormone signaling pathway	17		Haploinsufficient	5.53E-31	1.03E-28	31		-14	-1.14	ns	ns
Fanconi anemia pathway	18		Haploinsufficient	2.58E-30	4.82E-28	30		-12	-1.15	ns	ns
Epstein Barr virus infection	19		Haploinsufficient	8.01E-30	1.50E-27	39		-20	-1.07	ns	ns
Renal cell carcinoma	20		Haploinsufficient	5.13E-27	9.59E-25	40		-20	-1.07	ns	ns
Glucagon signaling pathway	21		Haploinsufficient	4.25E-26	7.95E-24	48		-27	-1.04	ns	ns
Chronic myeloid leukemia	22		Haploinsufficient	9.42E-26	1.76E-23		36	NA	0.99	ns	ns
Viral myocarditis	23		Haploinsufficient	1.36E-24	2.54E-22	68		-45	-0.93	ns	ns
p53 signaling pathway	24		Haploinsufficient	2.46E-24	4.60E-22	50		-26	-1.01	ns	ns
Protein processing in endoplasmic reticulum	25		Haploinsufficient	5.62E-24	1.05E-21	58		-33	-0.96	ns	ns
Viral carcinogenesis	26		Haploinsufficient	6.8E-23	1.27E-20	41		-15	-1.07	ns	ns
Biosynthesis of amino acids	27		Haploinsufficient	1.31E-22	2.45E-20	59		-32	-0.95	ns	ns
Transcriptional misregulation in cancer	28		Haploinsufficient	2.17E-22	4.06E-20	61		-33	-0.94	ns	ns
mTOR signaling pathway	29		Haploinsufficient	4.23E-22	7.91E-20	27		2	-1.19	ns	ns
Insulin secretion	30		Haploinsufficient	7.79E-22	1.46E-19	84		-54	-0.84	ns	ns
TNF signaling pathway	31		Haploinsufficient	1.07E-20	2.00E-18	34		-3	-1.12	ns	ns
AGE RAGE signaling pathway in diabetic complications	32		Haploinsufficient	1.27E-20	2.37E-18	33		-1	-1.13	ns	ns
Cytokine cytokine receptor interaction	33		Haploinsufficient	8.96E-20	1.68E-17	4		29	-1.64	0.001	ns
Apoptosis	34		Haploinsufficient	1.1E-19	2.06E-17	95		-61	-0.81	ns	ns
Herpes simplex infection	35		Haploinsufficient	1.96E-19	3.67E-17	80		-45	-0.87	ns	ns
RNA degradation	36		Haploinsufficient	3.84E-19	7.18E-17	20		16	-1.26	ns	ns
Starch and sucrose metabolism	37		Haploinsufficient	1.34E-18	2.51E-16	17		20	-1.29	ns	ns
PPAR signaling pathway	38		Haploinsufficient	1.88E-18	3.52E-16	102		-64	-0.77	ns	ns
Endometrial cancer	39		Haploinsufficient	2E-18	3.74E-16	94		-55	-0.81	ns	ns
Basal transcription factors	40		Haploinsufficient	3E-18	5.61E-16	92		-52	-0.82	ns	ns
Inositol phosphate metabolism	41		Haploinsufficient	3.74E-18	6.99E-16	43		-2	-1.07	ns	ns
Pancreatic cancer	42		Haploinsufficient	8.04E-18	1.50E-15	118		-76	-0.63	ns	ns
Fc epsilon RI signaling pathway	43		Haploinsufficient	1.08E-17	2.02E-15	99		-56	-0.80	ns	ns
Acute myeloid leukemia	44		Haploinsufficient	1.47E-17	2.75E-15	122		-78	-0.58	ns	ns
Vasopressin-regulated water reabsorption	45		Haploinsufficient	3.08E-17	5.76E-15	21		24	-1.26	ns	ns
NF kappa B signaling pathway	46		Haploinsufficient	4.48E-17	8.38E-15	93		-47	-0.82	ns	ns
Alcoholism	47		Haploinsufficient	5.31E-17	9.93E-15	91		-44	-0.82	ns	ns
Pantothenate and CoA biosynthesis	48		Haploinsufficient	5.41E-17	1.01E-14	83		-35	-0.85	ns	ns
Oxidative phosphorylation	49		Haploinsufficient	1.08E-16	2.02E-14	88		-39	-0.82	ns	ns
Phagosome	50		Haploinsufficient	1.58E-16	2.95E-14	116		-66	-0.65	ns	ns
SNARE interactions in vesicular transport	51		Haploinsufficient	2.17E-16	4.06E-14	37		14	-1.10	ns	ns
Colorectal cancer	52		Haploinsufficient	3.48E-16	6.51E-14	82		-30	-0.86	ns	ns
Toll like receptor signaling pathway	53		Haploinsufficient	1.43E-15	2.67E-13	98		-45	-0.81	ns	ns
RIG I like receptor signaling pathway	54		Haploinsufficient	4.32E-15	8.08E-13	56		-2	-0.98	ns	ns
Purine metabolism	55		Haploinsufficient	1.37E-14	2.56E-12	55		0	-0.99	ns	ns
Influenza A	56		Haploinsufficient	1.39E-14	2.60E-12	121		-65	-0.59	ns	ns
Metabolism of xenobiotics by cytochrome P450	57		Haploinsufficient	1.09E-09	2.04E-07	7		50	-1.57	0.027	ns
Chemical carcinogenesis	58		Haploinsufficient	2.27E-09	4.24E-07	5		53	-1.62	0.015	ns
Glycerophospholipid metabolism		1	Triploproficient	1.81E-63	3.38E-61		19	-18	1.18	ns	ns
Glycerolipid metabolism		2	Triploproficient	1.12E-58	2.09E-56		3	-1	1.64	0.018	ns
ECM receptor interaction		3	Triploproficient	2.13E-56	3.98E-54		4	-1	1.63	0.023	ns
Oxytocin signaling pathway		4	Triploproficient	1.42E-51	2.66E-49		7	-3	1.43	0.03	ns
Complement and coagulation cascades		5	Triploproficient	2.71E-47	5.07E-45		1	4	2.03	0	0.022
Small cell lung cancer		6	Triploproficient	5.3E-46	9.91E-44		6	0	1.60	0.021	ns
Focal adhesion		7	Triploproficient	1.73E-32	3.24E-30		23	-16	1.12	ns	ns
Wnt signaling pathway		8	Triploproficient	8.35E-32	1.56E-29	51		NA	-1.01	ns	ns
RNA transport		9	Triploproficient	3.41E-29	6.38E-27		29	-20	1.02	ns	ns
N-Glycan biosynthesis		10	Triploproficient	6.61E-29	1.24E-26		26	-16	1.07	ns	ns
Hippo signaling pathway		11	Triploproficient	1.42E-28	2.66E-26		45	-34	0.91	ns	ns
Cell adhesion molecules (CAMs)		12	Triploproficient	2.04E-28	3.81E-26		5	7	1.62	0.006	ns
MAPK signaling pathway		13	Triploproficient	3.13E-28	5.85E-26	75		NA	-0.89	ns	ns
Platelet activation		14	Triploproficient	3.6E-28	6.73E-26		9	5	1.34	ns	ns
Lysine degradation		15	Triploproficient	9.43E-28	1.76E-25		22	-7	1.13	ns	ns
Vascular smooth muscle contraction		16	Triploproficient	3.01E-27	5.63E-25		13	3	1.27	ns	ns
Peroxisome		17	Triploproficient	3.51E-27	6.56E-25		37	-20	0.98	ns	ns
Prolactin signaling pathway		18	Triploproficient	5.81E-27	1.09E-24		44	-26	0.91	ns	ns
Cell cycle		19	Triploproficient	3.94E-26	7.37E-24	70		NA	-0.91	ns	ns

Serotonergic synapse		20	Triploproficient	4.85E-26	9.07E-24		16	4	1.22	ns	ns
Pancreatic secretion		21	Triploproficient	9.67E-26	1.81E-23		15	6	1.23	ns	ns
Signaling pathways regulating pluripotency of stem cells		22	Triploproficient	1.74E-25	3.25E-23	110		NA	-0.71	ns	ns
Central carbon metabolism in cancer		23	Triploproficient	5.18E-25	9.69E-23	114		NA	-0.67	ns	ns
Dilated cardiomyopathy		24	Triploproficient	2.02E-24	3.78E-22		20	4	1.16	ns	ns
PI3K Akt signaling pathway		25	Triploproficient	2.09E-24	3.91E-22	71		NA	-0.91	ns	ns
Melanogenesis		26	Triploproficient	4E-24	7.48E-22	74		NA	-0.89	ns	ns
Gastric acid secretion		27	Triploproficient	5.17E-24	9.67E-22		21	6	1.14	ns	ns
Ribosome biogenesis in eukaryotes		28	Triploproficient	1.64E-22	3.07E-20	72		NA	-0.91	ns	ns
mRNA surveillance pathway		29	Triploproficient	1.84E-22	3.44E-20		11	18	1.33	ns	ns
Glycosylphosphatidylinositol (GPI) anchor biosynthesis		30	Triploproficient	1.94E-22	3.63E-20		24	6	1.11	ns	ns
Morphine addiction		31	Triploproficient	1.42E-21	2.66E-19		51	-20	0.83	ns	ns
Circadian entrainment		32	Triploproficient	1.42E-21	2.66E-19	73		NA	-0.89	ns	ns
Spliceosome		33	Triploproficient	2.17E-21	4.06E-19		31	2	1.02	ns	ns
Fc gamma R-mediated phagocytosis		34	Triploproficient	2.24E-21	4.19E-19		48	-14	0.88	ns	ns
Hypertrophic cardiomyopathy (HCM)		35	Triploproficient	1.06E-20	1.98E-18		18	17	1.19	ns	ns
Hepatitis B		36	Triploproficient	2.15E-20	4.02E-18		61	-25	0.69	ns	ns
Regulation of actin cytoskeleton		37	Triploproficient	2.27E-20	4.24E-18	89		NA	-0.82	ns	ns
Insulin resistance		38	Triploproficient	9.69E-20	1.81E-17	49		NA	-1.02	ns	ns
Adrenergic signaling in cardiomyocytes		39	Triploproficient	9.87E-20	1.85E-17		17	22	1.21	ns	ns
Cholinergic synapse		40	Triploproficient	1.81E-19	3.38E-17		40	0	0.95	ns	ns
Axon guidance		41	Triploproficient	2.25E-19	4.21E-17		10	31	1.34	ns	ns
Prostate cancer		42	Triploproficient	2.58E-19	4.82E-17	66		NA	-0.93	ns	ns
Arrhythmogenic right ventricular cardiomyopathy (ARVC)		43	Triploproficient	3.23E-19	6.04E-17		27	16	1.05	ns	ns
Retrograde endocannabinoid signaling		44	Triploproficient	2.43E-18	4.54E-16		28	16	1.03	ns	ns
Shigellosis		45	Triploproficient	3.01E-18	5.63E-16		50	-5	0.85	ns	ns
Basal cell carcinoma		46	Triploproficient	1.22E-17	2.28E-15	57		NA	-0.96	ns	ns
GABAergic synapse		47	Triploproficient	2.23E-17	4.17E-15	109		NA	-0.72	ns	ns
Chemokine signaling pathway		48	Triploproficient	2.7E-17	5.05E-15	8		NA	-1.56	0.007	ns
Gap junction		49	Triploproficient	3.26E-17	6.10E-15	26		NA	-1.19	ns	ns
Dopaminergic synapse		50	Triploproficient	3.43E-17	6.41E-15		47	3	0.90	ns	ns
HTLV-I infection		51	Triploproficient	9.94E-17	1.86E-14	60		NA	-0.94	ns	ns
Renin secretion		52	Triploproficient	3.42E-16	6.40E-14		12	40	1.30	ns	ns
Bacterial invasion of epithelial cells		53	Triploproficient	4.79E-16	8.96E-14	29		NA	-1.15	ns	ns
Bladder cancer		54	Triploproficient	1.75E-15	3.27E-13		34	20	1.01	ns	ns
Salivary secretion		55	Triploproficient	2.89E-15	5.40E-13		57	-2	0.75	ns	ns
cGMP PKG signaling pathway		56	Triploproficient	3.07E-15	5.74E-13		39	17	0.95	ns	ns
Malaria		57	Triploproficient	3.26E-15	6.10E-13	113		NA	-0.69	ns	ns
Proteoglycans in cancer		58	Triploproficient	3.33E-15	6.23E-13	87		NA	-0.82	ns	ns
Rap1 signaling pathway		59	Triploproficient	4.26E-15	7.97E-13	96		NA	-0.81	ns	ns
Pathogenic Escherichia coli infection		60	Triploproficient	7.03E-15	1.31E-12		8	52	1.40	ns	ns
Insulin signaling pathway		61	Triploproficient	1.44E-14	2.69E-12	85		NA	-0.84	ns	ns
Salmonella infection		62	Triploproficient	2.48E-14	4.64E-12		60	2	0.71	ns	ns
Oocyte meiosis		63	Triploproficient	2.81E-14	5.25E-12		56	7	0.75	ns	ns
Neurotrophin signaling pathway		64	Triploproficient	3.42E-14	6.40E-12	47		NA	-1.04	ns	ns
MicroRNAs in cancer		65	Triploproficient	4.76E-14	8.90E-12	24		NA	-1.23	ns	ns
ErbB signaling pathway		66	Triploproficient	5.28E-14	9.87E-12		46	20	0.90	ns	ns
Long-term depression		67	Triploproficient	5.8E-14	1.08E-11	35		NA	-1.11	ns	ns
Adherens junction		68	Triploproficient	5.91E-14	1.11E-11	106		NA	-0.75	ns	ns
Parkinson disease		69	Triploproficient	8.08E-14	1.51E-11	77		NA	-0.88	ns	ns
Estrogen signaling pathway		70	Triploproficient	8.1E-14	1.51E-11		30	40	1.02	ns	ns
Aldosterone synthesis and secretion		71	Triploproficient	8.11E-14	1.52E-11		49	22	0.85	ns	ns
Leukocyte transendothelial migration		72	Triploproficient	8.49E-14	1.59E-11	97		NA	-0.81	ns	ns
Glioma		73	Triploproficient	9.35E-14	1.75E-11	69		NA	-0.92	ns	ns
Amoebiasis		74	Triploproficient	1.11E-13	2.08E-11	67		NA	-0.93	ns	ns
Natural killer cell mediated cytotoxicity		75	Triploproficient	1.49E-13	2.79E-11	117		NA	-0.64	ns	ns
Regulation of lipolysis in adipocytes		76	Triploproficient	1.6E-13	2.99E-11		58	18	0.74	ns	ns
Inflammatory mediator regulation of TRP channels		77	Triploproficient	1.68E-13	3.14E-11	32		NA	-1.14	ns	ns
Alzheimer disease		78	Triploproficient	4.15E-13	7.76E-11		33	45	1.01	ns	ns
Phosphatidylinositol signaling system		79	Triploproficient	5.59E-13	1.05E-10		25	54	1.10	ns	ns
Melanoma		80	Triploproficient	7.34E-13	1.37E-10	81		NA	-0.87	ns	ns
Hedgehog signaling pathway		81	Triploproficient	7.69E-13	1.44E-10		41	40	0.94	ns	ns
Non alcoholic fatty liver disease (NAFLD)		82	Triploproficient	1.09E-12	2.04E-10	65		NA	-0.93	ns	ns
Pathways in cancer		83	Triploproficient	1.21E-12	2.26E-10	64		NA	-0.93	ns	ns
Endocytosis		84	Triploproficient	1.4E-12	2.62E-10	79		NA	-0.87	ns	ns
cAMP signaling pathway		85	Triploproficient	1.67E-12	3.12E-10	76		NA	-0.88	ns	ns
Longevity regulating pathway multiple species		86	Triploproficient	2.73E-12	5.11E-10	120		NA	-0.61	ns	ns
Cardiac muscle contraction		87	Triploproficient	2.76E-12	5.16E-10		43	44	0.92	ns	ns
TGF beta signaling pathway		88	Triploproficient	4.87E-12	9.11E-10		54	34	0.77	ns	ns
Legionellosis		89	Triploproficient	4.96E-12	9.28E-10		63	26	0.64	ns	ns
GnRH signaling pathway		90	Triploproficient	6.08E-12	1.14E-09	36		NA	-1.11	ns	ns
Choline metabolism in cancer		91	Triploproficient	7.11E-12	1.33E-09	100		NA	-0.79	ns	ns
Calcium signaling pathway		92	Triploproficient	8.86E-12	1.66E-09	38		NA	-1.08	ns	ns
Amino sugar and nucleotide sugar metabolism		93	Triploproficient	1.45E-11	2.71E-09	112		NA	-0.70	ns	ns
Sphingolipid signaling pathway		94	Triploproficient	1.76E-11	3.29E-09	44		NA	-1.06	ns	ns
ABC transporters		95	Triploproficient	2.07E-11	3.87E-09		42	53	0.93	ns	ns
Tight junction		96	Triploproficient	3.71E-11	6.94E-09	12		NA	-1.38	ns	ns
Primary immunodeficiency		97	Triploproficient	4.26E-11	7.97E-09		35	62	0.99	ns	ns
Hematopoietic cell lineage		98	Triploproficient	3.61E-10	6.75E-08		14	84	1.25	ns	ns
Olfactory transduction		99	Triploproficient	4.33E-06	8.10E-04		2	97	1.71	0	ns

Drug metabolism cytochrome P450			No Selection	ns	ns	18		NA	-1.29	ns	ns
Glutathione metabolism			No Selection	ns	ns	16		NA	-1.31	ns	ns
Nucleotide excision repair			No Selection	ns	ns	62		NA	-0.94	ns	ns
Osteoclast differentiation			No Selection	ns	ns		62	NA	0.66	ns	ns
Inflammatory bowel disease (IBD)			No Selection	ns	ns	46		NA	-1.05	ns	ns
Chagas disease (American trypanosomiasis)			No Selection	ns	ns	14		NA	-1.34	ns	ns
Leishmaniasis			No Selection	ns	ns	119		NA	-0.63	ns	ns
Pertussis			No Selection	ns	ns	115		NA	-0.66	ns	ns
Taste transduction			No Selection	ns	ns	52		NA	-1.01	ns	ns
Arachidonic acid metabolism			No Selection	ns	ns	45		NA	-1.05	ns	ns
Progesterone mediated oocyte maturation			No Selection	ns	ns	107		NA	-0.73	ns	ns
NOD like receptor signaling pathway			No Selection	ns	ns	53		NA	-1.00	ns	ns
Jak STAT signaling pathway			No Selection	ns	ns	111		NA	-0.70	ns	ns
Bile secretion			No Selection	ns	ns	104		NA	-0.77	ns	ns
Tuberculosis			No Selection	ns	ns	86		NA	-0.83	ns	ns
Fatty acid metabolism			No Selection	ns	ns	108		NA	-0.72	ns	ns
Thyroid hormone synthesis			No Selection	ns	ns	90		NA	-0.82	ns	ns
Cytosolic DNA-sensing pathway			No Selection	ns	ns		52	NA	0.83	ns	ns
Cocaine addiction			No Selection	ns	ns	105		NA	-0.75	ns	ns
Rheumatoid arthritis			No Selection	ns	ns	78		NA	-0.88	ns	ns
Toxoplasmosis			No Selection	ns	ns		32	NA	1.01	ns	ns
Amphetamine addiction			No Selection	ns	ns		59	NA	0.74	ns	ns
Glutamatergic synapse			No Selection	ns	ns	101		NA	-0.78	ns	ns
Measles			No Selection	ns	ns		64	NA	0.55	ns	ns
B cell receptor signaling pathway			No Selection	ns	ns		55	NA	0.76	ns	ns
Hepatitis C			No Selection	ns	ns	103		NA	-0.77	ns	ns
Phospholipase D signaling pathway			No Selection	ns	ns	63		NA	-0.94	ns	ns
Carbon metabolism			No Selection	ns	ns		65	NA	0.35	ns	ns
Ras signaling pathway			No Selection	ns	ns	25		NA	-1.21	ns	ns
T cell receptor signaling pathway			No Selection	ns	ns		53	NA	0.78	ns	ns

Supplementary Table 1: Direct comparison of HAPTRIG to GSEA on OV/KEGG analysis
GSEA was performed using identical parameters as used in HAPTRIG analysis presented here.
For p and q values, "ns" indicates $p>0.05$.

Autophagy

	Low					High				
	1	2	3	4	5	1	2	3	4	5
BLCA	ATG4B	GABARAP	ATG12	ATG16L1	MAP1LC3B	BECN1	ATG3	ATG7	PRKAA1	PIK3R4
BRCA	MAP1LC3B	GABARAPL2	GABARAP	ATG4B	ULK1	PRKAA1	MDM4	WWP1	CLN3	TSC2
CESC	GABARAP	ATG4B	ATG7	ATG12	MAP1LC3B	ATG3	PRKAA1	PIK3R4	PRKAA2	BECN1
COADREAD	GABARAP	PIK3C3	ATG12	HSP90AA1	ULK2	GABARAPL2	MAP1LC3B	ATG16L1	ATG4B	ATG4A
GBM	MAP1LC3B	GABARAPL2	GABARAP	ATG5	ATG13	ATG4D	PRKAA2	DNAJB1	CASP3	CASP8
HNSC	ATG7	ATG12	ATG4B	GABARAP	ATG10	ATG3	BECN1	GABARAPL1	PRKAA1	PIK3R4
KIRC	ATG7	ATG3	PIK3C3	BECN1	HSP90AA1	ATG12	ATG4B	ULK1	GABARAPL1	GABARAPL2
KIRP	HSPA5	ATG4C	MAP3K5	ULK3	DNAJB1	BECN1	GABARAPL2	GABARAP	MAP1LC3B	ATG3
LAML	GABARAP	ATG12	GABARAPL2	MAP1LC3B	ATG3	PRKAA2	WWP1	INS	DNAJB1	ATG16L2
LGG	PRKAA2	ATG4B	ATG3	ULK1	ATG12	ATG4D	GABARAPL1	CASP8	MAN2B1	ATG16L2
LIHC	GABARAP	MAP1LC3B	GABARAPL2	GABARAPL1	ATG13	BECN1	PRKAA1	ATG12	HSP90AB1	HSPA1A
LUAD	GABARAP	MAP1LC3B	ATG7	GABARAPL2	ATG12	BECN1	PRKAA1	PRKAA2	ULK1	HSP90AB1
LUSC	ATG7	GABARAP	ATG12	MAP1LC3B	GABARAPL2	ATG3	PRKAA1	GABARAPL1	PIK3R4	BECN1
OV	MAP1LC3B	BECN1	GABARAPL2	GABARAP	ATG12	GABARAPL1	ATG3	PRKAA1	PRKAA2	HSP90AB1
PAAD	GABARAP	PIK3C3	ATG7	ATG5	ATG12	ATG4B	MDM4	ATG4A	AGPS	TSC2
PRAD	MAP1LC3B	GABARAPL2	GABARAP	GABARAPL1	ATG5	WWP1	MDM4	ATG16L2	TSC2	IFNG
SARC	MAP1LC3B	GABARAPL2	ATG4B	GABARAP	ATG4A	PRKAA2	PRKAA1	ATG4D	HSP90AA1	DNAJB1
SKCM	MAP1LC3B	GABARAP	GABARAPL2	ATG5	ATG12	PRKAA1	HSPA1A	HSP90AB1	WWP1	MDM4
STAD	GABARAP	MAP1LC3B	ATG12	GABARAPL2	ATG7	PRKAA1	BECN1	HSP90AB1	WWP1	MDM4
THCA	UBE2L3	ATG4B	HSPA5	ATG16L1	ATG5	BECN1	ULK1	ATG12	GABARAPL1	GABARAP
UCEC	MAP1LC3B	GABARAPL2	GABARAP	BECN1	ATG12	PRKAA1	ATG3	PRKAA2	PIK3R4	GABARAPL1

ER Stress

	Low					High				
	1	2	3	4	5	1	2	3	4	5
BLCA	PARK2	HSPA8	STUB1	HSPA5	TP53	HSPA6	P4HB	NPLOC4	DNAJB11	NSFL1C
BRCA	HSPA8	FBXO6	TP53	PARK2	HSP90AA1	STUB1	HSPA6	YOD1	NPLOC4	HSPA1A
CESC	HSPA8	PARK2	TP53	CRYAB	MAPK8	DNAJB11	HSPA6	SEC61A1	HSPA1A	HSPA5
COADREAD	HSP90AA1	TP53	PARK2	FBXO6	LMAN1	HSPH1	HSP90AB1	UBE2D4	STUB1	HSPA1A
GBM	HSP90AA1	MAPK8	PARK2	HSPA8	STUB1	UBE2D4	DNAJB1	SMURF1	OS9	SEC61G
HNSC	HSPA8	CRYAB	MAPK9	MAPK8	FBXO6	HSPA5	HSP90AA1	DNAJB11	SEC61A1	HSPA6
KIRC	HSP90AA1	HSPA5	PARK2	FBXO6	HSPA1A	MAPK9	SEC24A	STUB1	UBE2D4	UBE2D2
KIRP	HSP90AA1	HSPA8	PARK2	UBE4B	MAP2K7	STUB1	TP53	P4HB	NPLOC4	AMFR
LAML	TP53	SEC24A	UBQLN1	STUB1	UBE2D4	HSPA8	FBXO6	CRYAB	HSP90AA1	HYOU1
LGG	HSP90AA1	FBXO6	PARK2	UBE4B	HSPA2	HSPA8	DNAJB1	UBE2D4	SMURF1	PRKCSH
LIHC	HSP90AA1	PARK2	TP53	HSPA8	FBXO6	HSPA1A	HSPA6	HSP90AB1	NPLOC4	YOD1
LUAD	PARK2	HSPA5	TP53	UBQLN1	MAP2K7	HSPA6	HSPA1A	NPLOC4	HSP90AB1	P4HB
LUSC	FBXO6	TP53	HSPA8	STUB1	MAPK9	DNAJB11	HSPA6	SEC61A1	NPLOC4	ERLEC1
OV	PARK2	HSPA5	STUB1	TP53	HSP90AA1	HSPA1A	HSPA6	DNAJB11	DNAJB1	NSFL1C
PAAD	PARK2	HSPA5	TP53	HSPA1A	FBXO6	HSPA6	UBE2D4	RFWD2	YOD1	SEC23A
PRAD	TP53	PARK2	UBE2J1	FBXO6	HSP90AA1	HSPA5	SEC61A1	UBE2D4	SMURF1	UBQLN1
SARC	HSPA8	TP53	MAPK8	CRYAB	HSPH1	HSPA5	UBQLN1	MAP2K7	DNAJB1	HSPA6
SKCM	HSPA8	PARK2	HSPA5	HSP90AA1	MAPK8	HSPA1A	HSP90AB1	HSPA6	HSPA1L	NPLOC4
STAD	PARK2	HSP90AA1	TP53	STUB1	MAP2K7	HSP90AB1	HSPA6	HSPA5	UBE2D4	HSPH1
THCA	HSPA5	ATF4	PARK2	UBQLN1	UBE2L3	HSP90AA1	STUB1	NPLOC4	HSPA6	P4HB
UCEC	HSPA5	HSPA8	TP53	AMFR	UBQLN1	HSPA6	HSPA1A	HSP90AB1	HSP90AA1	DNAJB1

Lysosome

	Low					High				
	1	2	3	4	5	1	2	3	4	5
BLCA	CTSD	PSAP	TP53	AP3M1	GGA1	CLTC	GGA3	AP1S1	ATR	SEC61A1
BRCA	FBXO6	AP1G1	AP1B1	GGA1	TP53	GGA2	CLTC	GGA3	CLN3	CTSE
CESC	CTSD	TP53	PSAP	HSPA8	CLTA	DNAJB11	GGA3	CLTC	ATR	SEC61A1
COADREAD	FBXO6	GGA1	TP53	AP4E1	SORT1	CTSA	AP1S1	NEU1	AP1G1	GGA2
GBM	GGA1	AP3M1	PSAP	CTSD	AP3S2	AP1S1	ATP6V0A4	GUSB	AP1G1	CTSA
HNSC	CTSD	FBXO6	AP3M1	ATG7	PSAP	CLTC	DNAJB11	AP1B1	ATR	AP1S1
KIRC	FBXO6	ATG7	GLB1	NPC2	NEU1	GGA2	CLTC	AP1G1	AP1S1	CLTB
KIRP	AP4E1	AP4S1	GBA	LGMN	AP4B1	CLTC	GGA3	GGA2	AP1G1	CLN3
LAML	AP1S1	AP1G1	TP53	CLTC	ATP6V0A4	GGA1	FBXO6	AP3M2	ATP6V0D2	CTSB
LGG	FBXO6	CTSD	SORT1	PPT1	PSAP	AP1S1	AP1G1	CLTC	ATP6V0A4	AP1M2
LIHC	FBXO6	TP53	CLTA	AP1G1	CTSB	CLTC	GGA3	CTSE	NEU1	CTSA
LUAD	GGA1	TP53	CLTA	MAN2B1	AP3M1	CLTC	GGA3	GGA2	CTSE	CTSA
LUSC	FBXO6	CTSD	TP53	PSAP	AP3M1	CLTC	AP1B1	GGA3	GGA1	M6PR
OV	GGA1	CLTC	AP1G1	CTSD	GGA2	DNAJB11	CTSA	SEC61A1	NEU1	ATR

PAAD	FBXO6	GGA1	CLTC	GGA3	TP53	AP1S1	CTSE	ATP6V0D2	CTSA	SEC61A1
PRAD	FBXO6	AP1G1	CLTC	TP53	AP1B1	ATP6V0D2	ATP6V1H	SEC61A1	ATP6V0A4	UBQLN1
SARC	CTSD	PSAP	AP1G1	TP53	CLTA	GGA3	FBXO6	CLTC	MAN2B1	GBA
SKCM	PSAP	CLTA	AP3M1	CTSD	TP53	CLTC	GGA1	GGA3	AP1S1	CTSA
STAD	GGA1	TP53	GGA2	AP4E1	AP1B1	CTSA	NEU1	AP1S1	ATP6V0D2	SEC61A1
THCA	GGA1	AP1B1	IGF2R	ATM	SORT1	CLTC	GGA3	GGA2	CLN3	MDM2
UCEC	AP1G1	GGA1	TP53	CTSD	AP4E1	CTSA	GGA3	DNAJB11	CTSE	NEU1

**Proteasome**	Low					High				
	1	2	3	4	5	1	2	3	4	5
BLCA	UBE2D2	PARK2	UBE2D3	UBE2D1	TRAF6	SKP2	UBR5	UBE2W	ANAPC11	TCEB1
BRCA	TP53	UBE2L3	PARK2	UBE2D3	WWP2	STUB1	UBE2I	RFWD2	UBE2W	UBR5
CESC	UBE2D3	PARK2	UBE2K	UBE2D1	TRAF6	RFWD2	SKP2	UBE2W	UBR5	UBE2C
COADREAD	UBE2D3	TP53	NEDD4L	UBE2L3	UBE3A	UBE2W	UBE2D4	UBE2C	UBR5	BIRC7
GBM	UBE2D1	PTEN	PARK2	UBE2L3	HUWE1	SMURF1	UBE2D4	FZR1	UBE2H	UBE2C
HNSC	UBE2D2	UBE2D3	UBE2E1	UBE2D1	UBA1	UBR5	UBE2W	SKP2	TCEB1	UBE2L3
KIRC	UBE2E1	PARK2	VHL	UBE2E2	HSP90AA1	UBE2D2	SKP1	UBE2B	MDM2	FBXW11
KIRP	UBE2L3	UBE4B	NEDD4L	HSP90AA1	SFN	BRCA1	STUB1	MDM2	UBE2I	TP53
LAML	UBE2D2	SKP1	UBE2B	TP53	SMURF1	UBE2W	UBR5	UBE2L3	DDB1	BIRC2
LGG	UBE2D1	UBE2D3	PARK2	UBA1	MDM2	SMURF1	FZR1	UBE2H	UBR5	UBE2D4
LIHC	UBE2D3	TP53	PARK2	HUWE1	UBE2G1	RFWD2	UBE2D2	UBR5	UBE2W	SKP2
LUAD	PARK2	FZR1	TP53	UBE2D2	UBE2D3	SKP2	RFWD2	UBE2W	UBR5	BRCA1
LUSC	UBE2D2	UBE2D3	UBE2D1	SKP1	MAP3K1	SKP2	UBE2L3	UBE2W	UBR5	SMURF1
OV	UBE2D3	FZR1	BRCA1	PARK2	UBE2L3	UBR5	RFWD2	SKP2	UBE2W	UBE2H
PAAD	PARK2	TP53	NEDD4L	UBE2L3	HUWE1	RFWD2	UBE2W	UBR5	UBE2D4	SMURF1
PRAD	TP53	PTEN	PARK2	UBE2L3	BRCA1	UBE2W	UBR5	TCEB1	UBE2D4	SMURF1
SARC	UBE2D1	TP53	XIAP	TRAF6	PTEN	MDM2	SKP2	UBE2D2	UBA1	FZR1
SKCM	UBE2D1	PARK2	UBE2D2	UBE2D3	PTEN	UBE2L3	RFWD2	UBE2W	SMURF1	UBE2H
STAD	UBE2D3	UBE2D2	PARK2	TP53	UBE2L3	UBE2W	UBR5	UBE2D4	UBE2C	SMURF1
THCA	UBE2L3	CHEK2	UBE2D1	ANAPC2	PARK2	UBE2D2	SKP1	RFWD2	STUB1	BRCA1
UCEC	UBE2D3	FZR1	TP53	UBE2D2	NEDD4	RFWD2	UBE2D1	UBE2W	UBR5	UBA1

**Peroxisome**	Low					High				
	1	2	3	4	5	1	2	3	4	5
BLCA	HSD17B4	ACSL4	CAT	TP53	SLC27A2	PEX19	PEX5	PEX12	PEX13	PEX10
BRCA	PEX14	SLC27A2	AGPS	TP53	HSD17B4	PEX19	PEX5	PEX11B	PEX2	ABCD2
CESC	HSD17B4	ACSL4	PEX7	SOD1	TP53	PEX19	PEX14	ABCD3	PEX5	PEX10
COADREAD	PEX14	HSD17B4	PRDX1	SOD1	ABCD3	PEX5	PEX19	PEX1	NOS2	PEX2
GBM	PEX7	ABCD1	PHYH	PEX14	PEX3	PEX19	PEX5	PEX1	GSTK1	PEX6
HNSC	HSD17B4	ACAA1	SOD1	SCP2	ACOX2	PEX5	PEX19	PEX2	ABCD1	PEX13
KIRC	PEX14	PEX3	ACAA1	PEX7	DHRS4	PEX5	HSD17B4	CAT	PEX19	AGPS
KIRP	PEX14	PEX3	SOD1	PRDX1	ABCD3	PEX5	NOS2	PEX12	ABCD1	AGPS
LAML	PEX5	HSD17B4	PEX1	GSTK1	CAT	PEX2	PEX19	SOD1	PEX14	PEX10
LGG	PEX14	ABCD3	PEX10	PRDX1	SCP2	PEX5	PEX1	GSTK1	CROT	PEX6
LIHC	PEX5	PEX14	SOD1	PEX7	PRDX1	PEX19	PEX11B	PEX26	PEX6	PEX1
LUAD	HSD17B4	PEX7	SLC27A2	ACAA1	SOD1	PEX19	PEX5	PEX12	PEX10	PEX11B
LUSC	HSD17B4	PEX14	ACAA1	SOD1	SCP2	PEX5	PEX19	PEX13	AGPS	EHHADH
OV	NOS2	PEX12	HSD17B4	PEX3	TP53	PEX5	PEX19	AGPS	PEX13	PEX11B
PAAD	PEX14	PEX7	SOD1	PEX3	PRDX1	PEX19	PEX5	PEX11B	PEX2	ABCD2
PRAD	PEX5	PEX14	AGPS	ABCD3	HSD17B4	PEX2	PEX1	PEX6	CYCS	PEX5L
SARC	PEX5	AGPS	ABCD1	ACSL4	TP53	PEX19	PEX14	PEX11B	PEX3	PRDX1
SKCM	PEX7	PEX3	HSD17B4	PHYH	SOD1	PEX19	PEX11B	PEX1	PEX6	PEX26
STAD	HSD17B4	SOD1	SCP2	ACAA1	PRDX1	PEX19	PEX5	PEX2	PEX13	PEX1
THCA	PEX26	SLC25A17	AGPS	SOD1	PEX6	PEX5	PEX19	PEX12	NOS2	HSD17B4
UCEC	HSD17B4	SLC27A2	TP53	NUDT7	NUDT12	PEX19	PEX5	PEX13	PEX11B	ABCD3

Blue= Significant allelic loss in this pathway in this cancer type
Red= Significant allelic gain in this pathway in this cancer type
Numbers indicate rank of most impactful gene, from 1 being most impactful to 5 being the 5th most impactful

Supplementary Table 2: The most positively and negatively impactful genes within HAPTRIG proteostasis networks
Genes were (scored with 1° interactions with other proteostasis pathways).
Pathways with significantly altered networks within a given cancer type are highlighted.

GISTIC2 Copy Number Alterations: Autophagy Genes

Cell Line	p53 mut	KRAS mut	BECN1	MAP1LC3B	ATG5	PIK3C3	GABARAPL2	ULK2	GABARAP	ATG10	ATG12	ATG13	ATG7	INS	ATG16L2
OVCAR3	R248Q	-	-1	-1	0	-1	-1	1	-2	0	0	1	0	0	2
OVCAR5	-	G12V	1	1	-1	1	-1	0	-1	0	1	-1	-1	-1	1
OVCAR8	X126_splice	P121H	1	1	-1	0	0	-1	-1	1	1	-1	1	-1	1
SKOV3	-	-	2	0	0	0	0	-1	-1	0	0	0	0	-1	0
IGROV1	Y126C	-	0	0	0	-1	0	0	0	0	0	-1	-1	0	0
A2780	-	-	0	0	0	0	-1	0	0	0	0	0	0	0	0

Cell Line	GABARAPL1	IFNG	ULK1	ATG14	ULK3	ATG4D	ATG4C	PRKAA2	ATG16L1	ATG4B	ATG3	PIK3R4	PRKAA1	ATG4A
OVCAR3	-1	0	0	1	-1	0	0	0	0	-1	2	2	1	0
OVCAR5	-1	1	-1	-1	1	1	0	0	0	-1	-1	1	0	-1
OVCAR8	0	0	1	1	0	1	1	1	0	0	1	1	1	0
SKOV3	-2	0	0	0	0	0	0	0	0	0	0	0	0	0
IGROV1	0	0	0	0	0	0	0	0	0	0	-1	0	0	-1
A2780	0	0	0	0	0	0	0	0	0	0	0	0	0	0

Cell Line	IFNA1	IFNA10	IFNA13	IFNA14	IFNA16	IFNA17	IFNA2	IFNA21	IFNA4	IFNA5	IFNA6	IFNA7	IFNA8
OVCAR3	1	1	1	1	1	1	1	1	1	1	1	1	1
OVCAR5	-1	-1	-1	-1	-1	-1	-1	-1	-1	-1	-1	-1	-1
OVCAR8	0	0	0	0	0	0	0	0	0	0	0	0	0
SKOV3	-1	-1	-1	-1	-1	-1	-1	-1	-1	-1	-1	-1	-1
IGROV1	0	0	0	0	0	0	0	0	0	0	0	0	0
A2780	0	0	0	0	0	0	0	0	0	0	0	0	0

Unknown Genetics: U373, LPPDOV, OVCAR10

Supplementary Table 3: Autophagy gene status of cell lines studied

Gain/loss scores from the Cancer Cell Line Encyclopedia as curated by the cBioPortal were used to determine copy number aberrations. KRAS and TP53 mutations are also shown.

Year	Study PMID	Study Title	Pathway Finding	Gene Findings (+ Regulation)	Gene Findings (- Regulation)	Gene Findings (Mutation)	In Vitro test	In Vivo Test
(Current study)		Haploinsufficiency Networks Identify Targetable Patterns of Allelic Deficiency in Low Mutation Ovarian Cancer	Autophagy	CTSD, PEX5, PCAT1/2, many more	MAP1LC3B, BECN1	N/A	10+ models	3 models
2016	27372738	Integrated Proteogenomic Characterization of Human High-Grade Serous Ovarian Cancer	HR, JAK/STAT, SRF, PDGFRb	N/A	N/A	N/A	N/A	N/A
2015	25888305	A network model for angiogenesis in ovarian cancer	Angiogenesis	NFKB1, ARID3A, SOX5, TFAP2A, NKX2-5, PRRX2	AHR, SPIB, MZF1, BRCA1	N/A	N/A	N/A
2015	25710373	Spatial and Temporal Heterogeneity in High-Grade Serous Ovarian Cancer: A Phylogenetic Analysis	Clonal expansions	2q, 3p, Chr10	NF1	TP53, BRCA1/2, NF1, APC	N/A	N/A
2015	26017449	Whole-genome characterization of chemoresistant ovarian cancer	BRCA, MDR	CCNE1, MDR1, ABCB1	RB1, NF1, RAD51B, PTEN	BRCA1, BRCA2	N/A	N/A
2015	25581432	Gene expression analysis identifies global gene dosage sensitivity in cancer	DNA stability	MYC, CCNE1, PIK3CA, BIRC5	TP53, CDKN2A, RB1, BRCA1, BRCA2, ATM	N/A	N/A	N/A
2014	24448499	Integrated analysis of germline and somatic variants in ovarian cancer	Fanconi Anemia, MAPK, MLL, DNA repair	N/A	BRCA, FANC, ATR, others	NF1, KRAS, others	N/A	N/A
2014	24071852	Pan-cancer patterns of somatic copy number alteration	Whole-genome-duplication	CCND1, EGFR, MYC, ERBB2, CCNE1, MCL1, MDM2, WHSC1L1, TERC, TERT, RMRP	ATM, NOTCH, PPP2RA, PTTG11P, FOXK2, LINC00290, ERRF11, FOXC1	N/A	N/A	N/A
2013	24071851	Emerging landscape of oncogenic signatures across human cancers	Cell cycle, BRCA	MYC, CCNE1	TP53, BRCA	TP53, others	N/A	N/A
2013	23822816	Large-scale integrative network-based analysis identifies common pathways disrupted by copy number alterations across cancers	TGFb, MAPK, MYC	MYC, TRIB1, E2F3, AURKA	NCOR1, PPP2RA, CLU, MAP2K4	N/A	N/A	N/A
2011	21720365	Integrated genomic analyses of ovarian carcinoma	RB, RAS, HR, NOTCH, FOXM1	MYC, CXCL10/11, CXCR3, HMGA2, SOX11, MCM2, PCNA, MUC16, MUC1, SLPI, HOX	MUC1, MUC16	TP53, NF1, BRCA1, BRCA2, RB1, CDK12, CSMD3, FAT3, GABRA6	N/A	N/A
2009	19193619	Integrated genome-wide DNA copy number and expression analysis identifies distinct mechanisms of primary chemoresistance in ovarian carcinomas	Cell cycle, ECM deposition	CCNE1, ERBB2, MUC1, MYC	BRCA1, IGF2R	N/A	N/A	N/A

Supplementary Table 4: Notable pathway analyses performed for OV.
